# Imidazoline- and Benzamidine-Based Trypanosome Alternative Oxidase Inhibitors: Synthesis and Structure–Activity Relationship Studies

**DOI:** 10.1021/acsmedchemlett.1c00717

**Published:** 2022-01-28

**Authors:** David Cisneros, Eduardo J. Cueto-Díaz, Tania Medina-Gil, Rebecca Chevillard, Teresa Bernal-Fraile, Ramón López-Sastre, Mustafa M. Aldfer, Marzuq A. Ungogo, Hamza A. A. Elati, Natsumi Arai, Momoka Otani, Shun Matsushiro, Chiaki Kojima, Godwin U. Ebiloma, Tomoo Shiba, Harry P. de Koning, Christophe Dardonville

**Affiliations:** † Instituto de Química Médica, IQM−CSIC, Juan de la Cierva 3, E-28006 Madrid, Spain; ‡ Graduate School of Science and Technology, Department of Applied Biology, Kyoto Institute of Technology, Kyoto 606-8585, Japan; § Institute of Infection, Immunity and Inflammation, College of Medical, Veterinary and Life Sciences, University of Glasgow, Glasgow G12 8TA, United Kingdom; ∥ School of Health and Life Sciences, Teesside University, Middlesbrough TS1 3BX, United Kingdom

**Keywords:** Trypanosome alternative oxidase inhibitor, *Trypanosoma brucei*, benzamidine, imidazoline, glycolysis

## Abstract

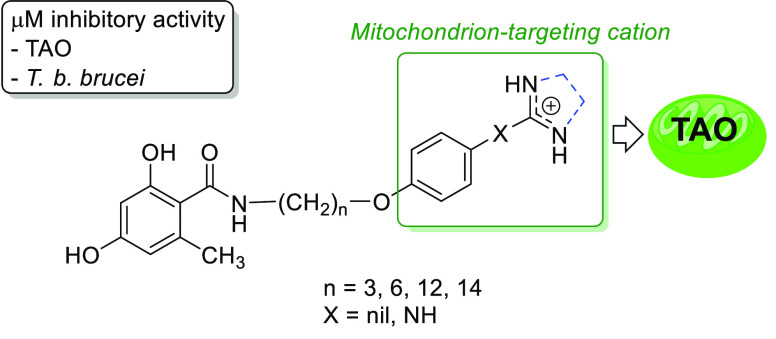

The trypanosome alternative oxidase (TAO), a mitochondrial enzyme involved in the respiration of the bloodstream form trypomastigotes of *Trypanosoma brucei*, is a validated drug target against African trypanosomes. Earlier series of TAO inhibitors having a 2,4-dihydroxy-6-methylbenzoic acid scaffold (“head”) and a triphenylphosphonium or quinolin-1-ium cation as a mitochondrion-targeting group (“tail”) were shown to be nanomolar inhibitors in enzymatic and cellular assays. We investigated here the effect of different mitochondrion-targeting cations and other scaffold modifications on the in vitro activity of this class of inhibitors. Low micromolar range activities were obtained, and the structure–activity relationship studies showed that modulation of the tail region with polar substituents is generally detrimental to the enzymatic and cellular activity of TAO inhibitors.

African trypanosomes (*Trypanosoma brucei* sp.) are protozoan parasites that cause sleeping sickness (human African trypanosomiasis, HAT) in sub-Saharan Africa. Bloodstream form (BSF) trypomastigotes of *T. brucei* possess a unique energy metabolism as they only depend on glycolysis for energy supply.^1,2^ In the absence of a functional oxidative phosphorylation pathway, they use the trypanosome alternative oxidase (TAO) to reoxidize the NADPH that is formed during glycolysis.^3^ TAO is essential for the respiration of BSF trypomastigotes,^3^ is conserved among trypanosome subspecies,^4^ has no counterpart in mammalian cells, and has been validated as a drug target in trypanosomes.^5^

The localization of TAO at the interface of the inner mitochondrial membrane^6,7^ has inspired the development of potent 4-hydroxybenzoate- and 4-alkoxybenzaldehyde-based inhibitors that hold a lipophilic cation as the mitochondrion-targeting moiety.^8−10^ In particular, 2,4-dihydroxy-6-methylbenzoate derivatives were nanomolar range TAO inhibitors showing in vitro and in vivo trypanocidal activity in a mouse model of *T. b. rhodesiense* infection (Chart 1A).^9^ Mitochondrial localization of this class of inhibitors was confirmed by live-cell imaging with fluorescent analogues.^11^

**Chart 1:**
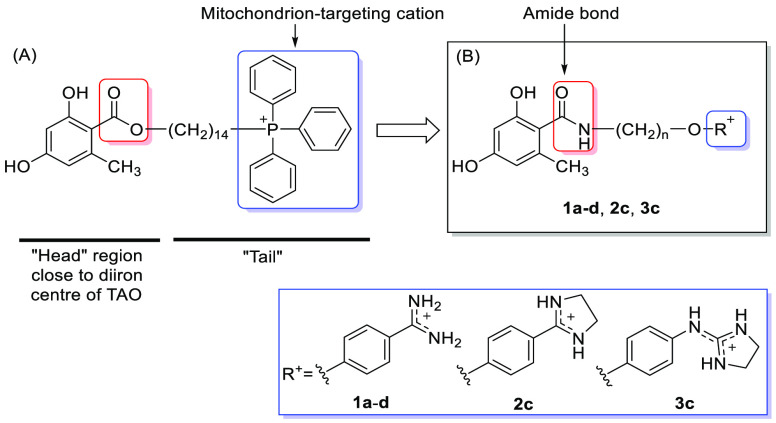
(A) Example of Previously Reported Benzoate TAO Inhibitors with a Triphenylphosphonium Mitochondrion-Targeting Cation^9^ and (B) Structural Modifications Studied in This Work

In the current study, new analogues of the benzoate lead compound were synthesized to extend the structure–activity relationship (SAR) of this class of TAO inhibitors. The first modification was the replacement of the ester bond by a more metabolically stable amide bond. Grady et al.^12^ showed that this structural modification produced inhibitors that were more soluble and more stable to serum hydrolases in vivo than the benzoate counterparts.^5^ Second, different cationic groups were tested in place of the bulky triphenylphosphonium (TPP^+^) and quinolin-1-ium cations that were used in the previous series, in which positive charge is highly delocalized.^8,9^

We observed previously, in model structures of TPP^+^-linked inhibitors binding to TAO, that the methylene linker (tail) engaged in hydrophobic interactions with the hydrophobic region of the enzyme cavity, whereas the large TPP cation extended outward into the solvent.^9^ In the present work, we tested the benzamidinium (**1a**–**d**), 2-phenylimidazolin-3-ium (**2c**), and 2-(phenylamino)imidazolin-3-ium (**3c**) cations as less bulky surrogates of TPP^+^ (Chart 1). Compounds containing these cationic groups, which are found in many trypanocidal drugs (e.g., pentamidine, diminazene) and investigational compounds, are known to strongly accumulate in the mitochondrial matrix of trypanosomes, against considerable concentration gradients.^13−19^ We hypothesized that smaller cations would insert themselves deeper into the enzyme cavity to promote favorable interactions of the 2,4-dihydroxy-6-methylbenzoic head with the enzyme active site. With the previous 4-hydroxybenzoate series, a methylene linker of less than C-14 between the TPP or quinolin-1-ium cation and the head region was detrimental to TAO inhibition.^9,10^ However, the imidazoline- and benzamidine-based cations used in this study are structurally different (i.e., shape, size, and electronic properties) to these cations and may present a different SAR. Hence, a methylene linker covering a wide range of lengths between the 2,4-dihydroxy-6-methylbenzoic scaffold and the cationic group were tested ((CH_2_)_*n*_, *n* = 3, 6, 12, 14). All of the compounds were assayed against recombinant TAO enzyme and wild-type (WT) and drug-resistant *T. b. brucei* strains.

## Results and Discussion

**Scheme 1:**
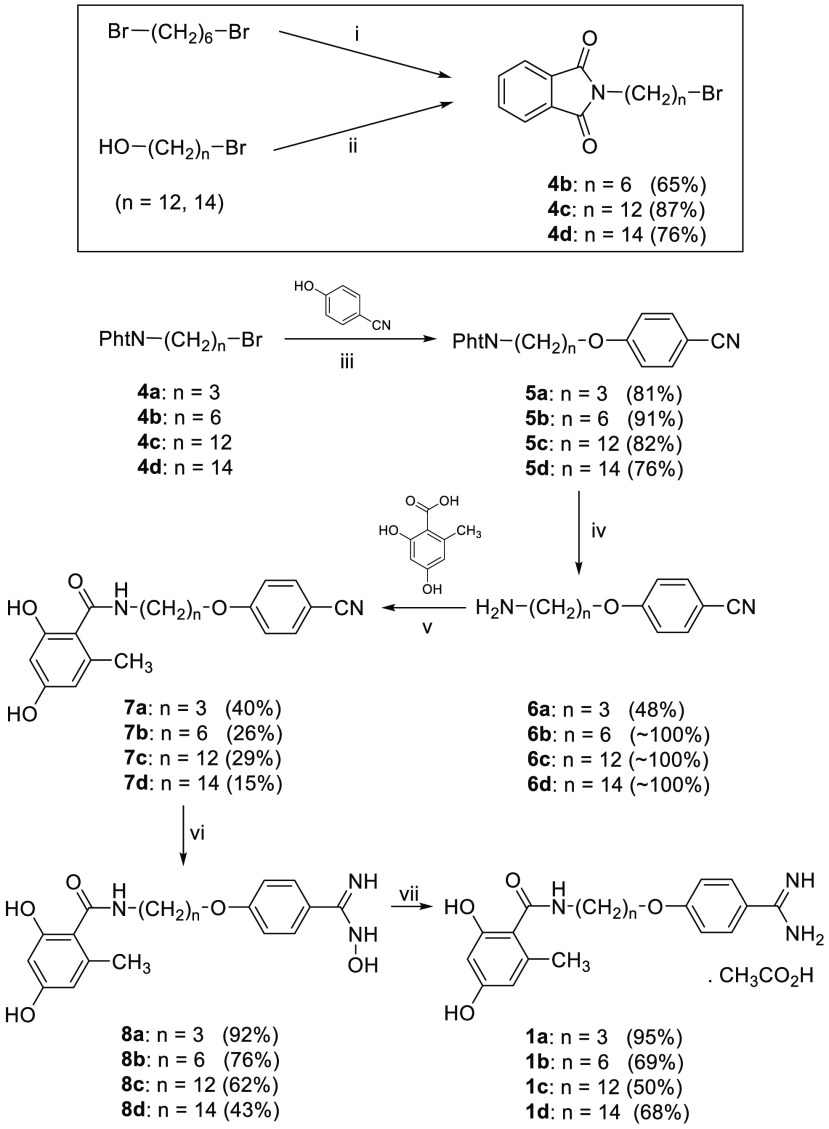
Synthesis of Benzamidine Derivatives **1a**–**d** Reagents and conditions. (i) PhtN^–^K^+^, DMF, rt, 20 h; (ii) PhtNH, Ph_3_P, DIAD, THF, 0 °C then rt, 20 h; (iii) K_2_CO_3_, CH_3_CN, 80 °C, 24 h; (iv) N_2_H_4_·H_2_O, EtOH, 80 °C, 12 h; (v) for **7a**, **7c**, and **7d**, EDC·HCl, DMAP, CH_3_CN, 80 °C, 20 h; for **7b**, PyBOP, DIPEA, DMF, rt, 18 h; (vi) NH_2_OH·HCl, ^*t*^BuOK, DMSO, rt, 4 days; (vii) (1) Ac_2_O, AcOH, 15 min, (2) H_2_, 5% Pd–C, AcOH, rt, 12 h.

The benzamidine derivatives with methylene linkers of 3, 6, 12, and 14 units (**1a**–**d**) were synthesized in five steps from the corresponding *N*-(*n*-bromoalkyl)phthalimide **4a**–**d** (Scheme 1).

Compound **4a** was commercially available, whereas **4b**–**d** were synthesized, as shown in Scheme 1. A reaction of potassium phthalimide with an excess of 1,6-dibromohexane yielded **4b**. Compounds **4c** and **4d** were obtained in good yields from phthalimide and 12-bromododecan-1-ol^20^ or 14-bromotetradecan-1-ol^8^ using the Mitsunobu protocol. Reaction of **4a**–**d** with 4-cyanophenol and K_2_CO_3_ generated ethers **5a**–**d**, which were converted to amines **6a**–**d** using hydrazine monohydrate. The coupling of amines **6a**–**d** with orsellinic acid was achieved with EDC hydrochloride and a catalytic amount of DMAP to give **7a**, **7c**, and **7d** in low to moderate yields (15–40%). For the synthesis of **7a** and **7b**, another coupling agent (PyBOP) was tried, but no improvement of yield was observed (14 and 26%, respectively). Of note, the yield of this amide coupling seemed to decrease with the methylene chain length of the amine, reflecting more complex reaction crudes and, possibly, solubility issues (e.g., amine **6d** was only soluble in hot acetonitrile).

**Scheme 2:**
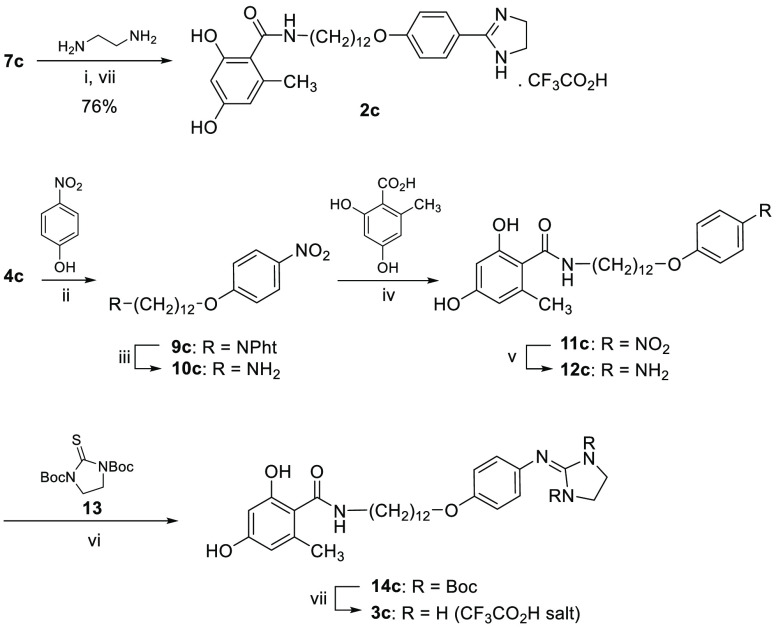
Synthesis of Imidazoline (**2c**) and 2-Aminoimidazoline (**3c**) Derivatives Reagents and conditions: (i) P_2_S_5_, 1,2-ethylenediamine, sealed tube, 120 °C, 2 h; (ii) K_2_CO_3_, CH_3_CN, 80 °C; (iii) N_2_H_4_·H_2_O, EtOH, 80 °C; (iv) EDC·HCl, DMAP, CH_3_CN, CH_2_Cl_2_, 80 °C; (v) H_2_, MeOH, Pd–C 5%; (vi) HgCl_2_, Et_3_N, DMF, 0 °C to rt; (vii) CH_2_Cl_2_, TFA, 0 °C.

Benzamidine synthesis was achieved in a two-step process involving the formation of benzamidoximes **8a**–**d** followed by the catalytic hydrogenation of intermediate benzamidoximes in acetic acid/acetic anhydride to yield **1a**–**d**.^21^ Imidazoline derivative **2c** was synthesized in good yield (76%) by reaction of the cyano derivative **7c** with ethylenediamine/P_2_S_5_ at 120 °C in a sealed tube (Scheme 2). The 2-aminoimidazoline analogue **3c** was obtained in two steps from the amino precursor **12c** using di-*tert*-butyl 2-thioxoimidazolidine-1,3-dicarboxylate (**13**) following a known protocol.^13,22^ Compound **12c** was synthesized in four steps from **4c** and 4-nitrophenol following the same route as described for the synthesis of **7a**–**d** (Scheme 2).

**Scheme 3:**
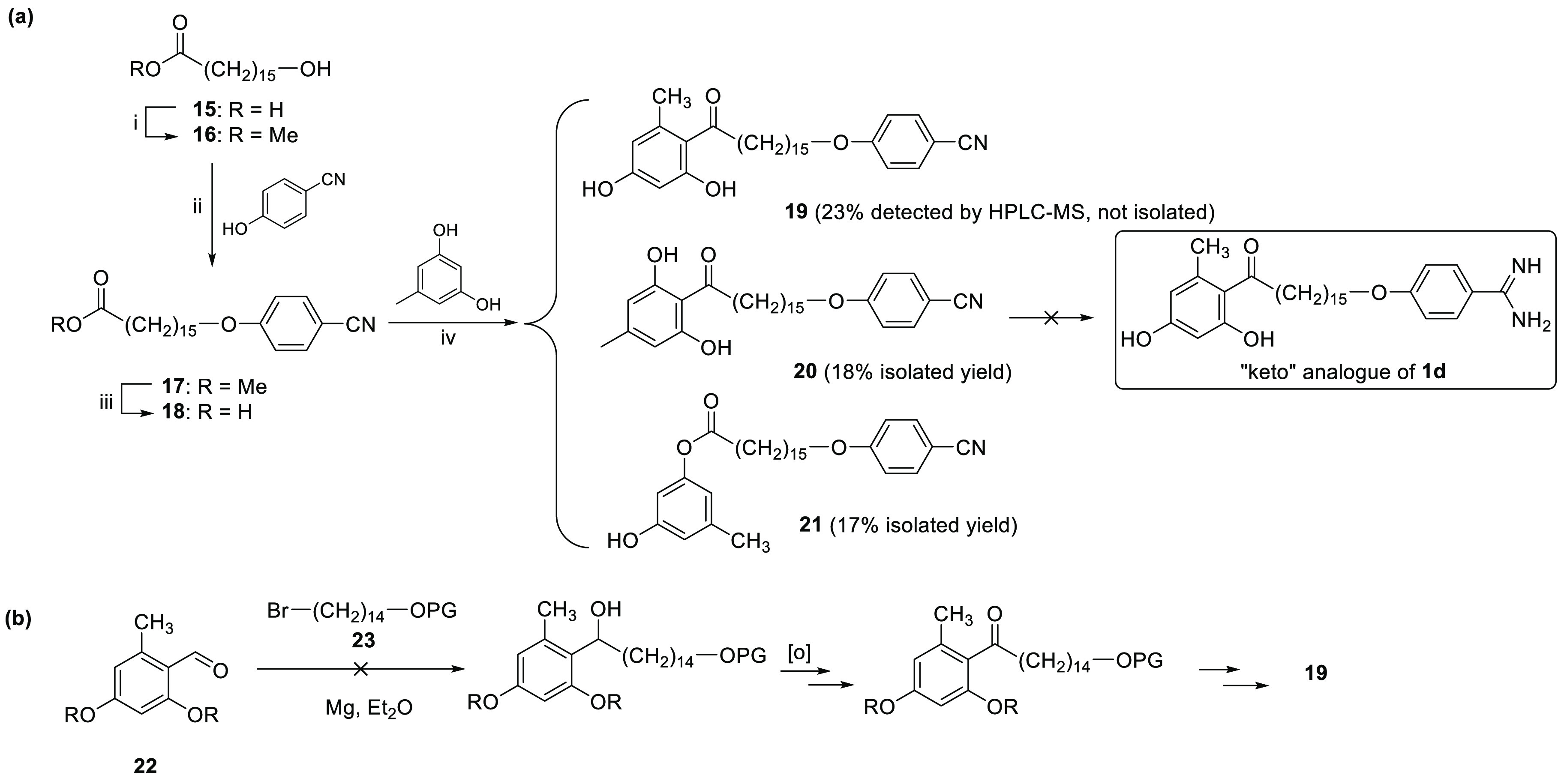
(a) Synthesis of Derivatives **19**–**21** and (b) Attempted Route toward Compound **19** Reagents and conditions: (i) MeOH, TsOH–H_2_O, rt, 21 h, (96%); (ii) PPh_3_, DIAD, THF, 0 °C to rt, 4 days (58%); (iii) LiOH·H_2_O, THF/MeOH/H_2_O (2/1/1), rt (94%); (iv) AlCl_3_, 1,2-dichloroethane, 100 °C, 24 h.

We sought to understand the role of the amide bond in the binding to the TAO active site. To do so, we tried to prepare the “keto” analogue of **1d**, with a carbonyl bond linking the methylene chain to the 5-methylresorcinol scaffold instead of an amide bond (Scheme 3a). 16-(4-Cyanophenoxy)hexadecanoic acid **18** was synthesized in three steps from 16-hydroxyhexadecanoic acid **15**. Friedel–Crafts acylation of 5-methylresorcinol with **18** using AlCl_3_ gave a 23:20:57 mixture of three isomers **19**/**20**/**21** as detected by HPLC-MS. Compounds **20** and **21** were isolated (18 and 17% yield, respectively) and characterized by ^1^H and ^13^C NMR. However, we were unable to isolate the desired isomer **19** from the mixture due to very similar chromatographic behavior with **20** and **21**. Attempts at the synthesis with a different Lewis acid (i.e., BF_3_–Et_2_O) led to the formation of more complex reaction mixtures. As an alternative route, the reaction of 2,4-dihydroxy-6-methylbenzaldehyde **22** with the Grignard reagent of **23** was tried several times using different conditions but without success (Scheme 3b). Hence, attempts to obtain sufficiently pure **19** were dropped. Nevertheless, the biological activity of intermediates **20** and **21**, useful for SAR studies, is reported in Table 1.

**Table 1:** In Vitro Activity of Amidines (**1a**–**d**), Hydroxyamidines (**8a**–**d**), Imidazolines (**2c**, **3c**, and **14c**), and Synthetic Intermediates (**7a**–**d**, **11c**, **12c**, **20**, and **21**)

		*T. b. brucei*					cytotoxicity		rTAO^g^ % inhibition at 40 μM	rTAO
		EC_50_ (μM)					CC_50_ (μM)			IC_50_ (μM)
cmpd	*n*	WT^a^	B48^b^	RF^c^	AQP1-3 KO^d^	RF^c^	HEK^e^	SI^f^		
**1a**	3	>100	>100		nd		>200		48%	>40^j^
**1b**	6	15.5 ± 0.6	28.0 ± 0.5	1.8	17.4 ± 0.9	1.1	>200	>12.9	12.5%	>40
**1c**	12	3.3 ± 0.2	3.6 ± 0.1	1.1	nd		43.5 ± 5.4	13.2	39.3%	>40
**1d**	14	18.6 ± 1.1	28.9 ± 5.2	1.6	nd		>200	10.7	31.4%	>40
**2c**	12	1.7 ± 0.3	nd		2.9 ± 0.4	1.7	67.2 ± 0.8^h^	39.1	10.0%^i^	>10
**3c**	12	2.7 ± 0.4	nd		3.92 ± 0.03	1.4	>100^h^	>36.5	46.3%^i^	22.5 ± 0.3
**7a**	3	37.2 ± 3.4	50.8 ± 2.7	1.4	nd		>200	>5.3	28.8%	>40
**7b**	6	30.4 ± 0.4	27.7 ± 2.0	0.9	30.5 ± 0.7	1.0	>200	6.6	96.2%	1.5 ± 0.1
**7c**	12	15.6 ± 0.7	15.9 ± 0.7	1.0	nd		57.1 ± 0.1	3.7	27.4%	>40
**7d**	14	14.8 ± 0.5	15.7 ± 0.5	1.1	nd		56.8 ± 0.2	3.8	89.7%	16.4 ± 0.7
**8a**	3	>100	>100		nd		>200		14.1	>40
**8b**	6	19.5 ± 1.0	36.0 ± 1.8	1.9	21 ± 1	1.1	76.8 ± 7.2	3.9	–14.9%	>40
**8c**	12	8.4 ± 1.1	9.1 ± 0.5	1.1			108.9 ± 1.6	13	44.8	>40
**8d**	14	29.0 ± 2.2	27.1 ± 2.4	0.9	nd		>200	6.9	–3.4%	>40
**11c**	12	9.5 ± 0.5	nd		10.2 ± 0.7	1.1	>100^h^	>10.5	53.8%^i^	30.0 ± 1.5
**12c**	12	20.3 ± 1.3	nd		29.6 ± 2.2	1.5	>100^h^	>4.9	nd	nd
**14c**	12	10.4 ± 0.7	nd		15.7 ± 0.3	1.5	>100^h^	>9.6	nd	nd
**20**	15	5.8 ± 0.7	nd		8.2 ± 0.9	1.4	>100^h^	>17.1	13.4%^i^	>10
**21**	15	>100	nd		>100		>100^h^		30.0%^i^	>10
diminazene		0.095 ± 0.011	0.107 ± 0.019	1.1						
pentamidine		0.004 ± 0.001	0.208 ± 0.021	49.5	0.046 ± 0.003	10.8				
phenylarsine oxide							0.9 ± 0.1			
ascofuranone									100%	

a Bloodstream form trypomastigotes of *T. b. brucei* strain 427 (*n* = 3).

b *T. b. brucei* strain resistant to pentamidine (*n* = 3).

c Resistance factor relative to WT.

d *T. brucei* cell line from which all aquaporins were knocked out (*n* = 3).

e Cytotoxicity on human embryonic kidney cells (*n* = 3).

f Selectivity index (SI) = CC_50_/EC_50_ (WT).

g Purified recombinant trypanosome alternative oxidase (ΔMTS-TAO)^9^ from *T. b. brucei* (*n* = 3); compound concentration = 40 μM.

h *n* = 2.

i Compound tested at 10 μM concentration.

j No reliable IC_50_ could be obtained for inhibitors with less than 40% single-point inhibition as a sigmoidal curve could not be generated.

### Biology

The trypanocidal activity of compounds **1a**–**d**, **2c**, **3c**, **7a**–**d**, and **8a**–**d** and synthetic intermediates **11c**, **12c**, **14c**, **20**, and **21** against wild-type (s427) and drug-resistant strains of *T. b. brucei* (i.e., B48, AQP1-3 KO) was determined in vitro using a resazurin-based assay.^8,9^ In general, a methylene linker of 12 carbons gave the lowest EC_50_ values against *T. brucei* (compare **1a**–**d**/ **7a**–**d**/ **8a**–**d**). Target compounds **1c**, **2c**, and **3c** were the most effective compounds of the series with EC_50_ values of <4 μM against *T. brucei* (Table 1). Among them, the 2-phenylimidazolin-3-ium derivative **2c** was marginally more active with EC_50_ = 1.72 μM. This finding was in agreement with previous reports on TAO inhibitors showing that a decrease in efficacy against *T. b. brucei* growth inhibition was observed as chain length decreased.^8,9,23^ Hence, compounds with short linkers (C-3) were poorly active (**7a**) or inactive (**1a**, **8a**) against *T. brucei*. Substituents in the *para* position of the phenoxy group affected the trypanocidal activity in the following order: 2-phenylimidazolin-3-ium (**2c**) > 2-(phenylamino)imidazolin-3-ium (**3c**) ≈ benzamidinium (**1c**) > *N*-hydroxyamidine (**8c**) ≈ 4-NO_2_ (**11c**).

Apparently, the effect of changing the amide connecting group in **7d** with a keto bond (**20**) was favorable for anti-*T. brucei* activity, as shown by the 2.5-fold lower EC_50_ of **20** (5.8 μM) versus **7d** (14.8 μM). However, because **7d** and **20** are slightly different isomers (2,4-dihydroxy-6-methyl and 2,6-dihydroxy-4-methyl, respectively), an isomer-dependent effect cannot be ruled out. Wild-type and drug-resistant strains showed virtually the same susceptibility toward these compounds (within 2-fold difference), indicating that the compounds, unlike some other benzamidines such as pentamidine,^24^ are not dependent on aquaporins, or on the aminopurine transporter TbAT1, for uptake by *T. brucei*. Cytotoxicity against HEK cells was low, resulting in selectivity indexes of >10 for **1b**–**d** to >36.5 for **2c** and **3c**.

The compounds were screened at a single concentration (either 10 or 40 μM) as inhibitors of the ubiquinol oxidase activity of purified ΔMTS-TAO. The IC_50_ values of the compounds displaying the best percentage of inhibition were also determined (Table 1).^9^ In general, the benzamide derivatives reported here were poor TAO inhibitors, with IC_50_ values in the micromolar range compared to the nanomolar range inhibitors reported previously.^8,9^ The best inhibitors were the uncharged 4-cyanophenoxy analogues **7b** (IC_50_ = 1.52 μM, C-6 methylene linker) > **7d** (IC_50_ = 16.4 μM, C-14 methylene linker). More polar substituents in the *para* position of the phenoxy group such as 2-(phenylamino)imidazolin-3-ium (**3c**) or 4-NO_2_ (**11c**) gave less potent inhibitors (IC_50_ = 22.5 and 30 μM, respectively). These SAR results regarding TAO activity were consistent with previous work showing that the introduction of polar substituents in the tail region of TAO inhibitors is not well-tolerated, leading to a strong decrease in inhibitory potency.^23^ This effect seems to be counterbalanced when lipophilic cations such as TPP^+^ or quinolin-1-ium are used, but in that case, the linker length in the tail region must be long enough (≥C-14) to allow the bulky TPP cation to remain outside the enzyme active site, giving rise to low nanomolar TAO inhibitors.^5,9^ For the benzamidine-based TAO inhibitors **1a**–**d**, a linkage of 14 methylene units did not improve TAO inhibition versus the C-12 linker, as opposed to the previous series having a quinolinium or TPP cations.^9^ In that case, the aromatic moieties of TPP and quinolinium cations interact with the surface of the enzyme, and the linker length must give the flexibility to the aromatic ring to orient itself optimally. Apparently, such interactions may not happen for the benzamidine compounds reported here.

Unfortunately, our efforts to isolate pure keto analogue **19**, which would have informed about the effect of the amide bond on TAO inhibitory activity, were unsuccessful. However, the lack of TAO inhibition by the structurally close analogue **20** seems to indicate that the keto connection is not substantially superior to the amide linkage.

**Figure 1:**
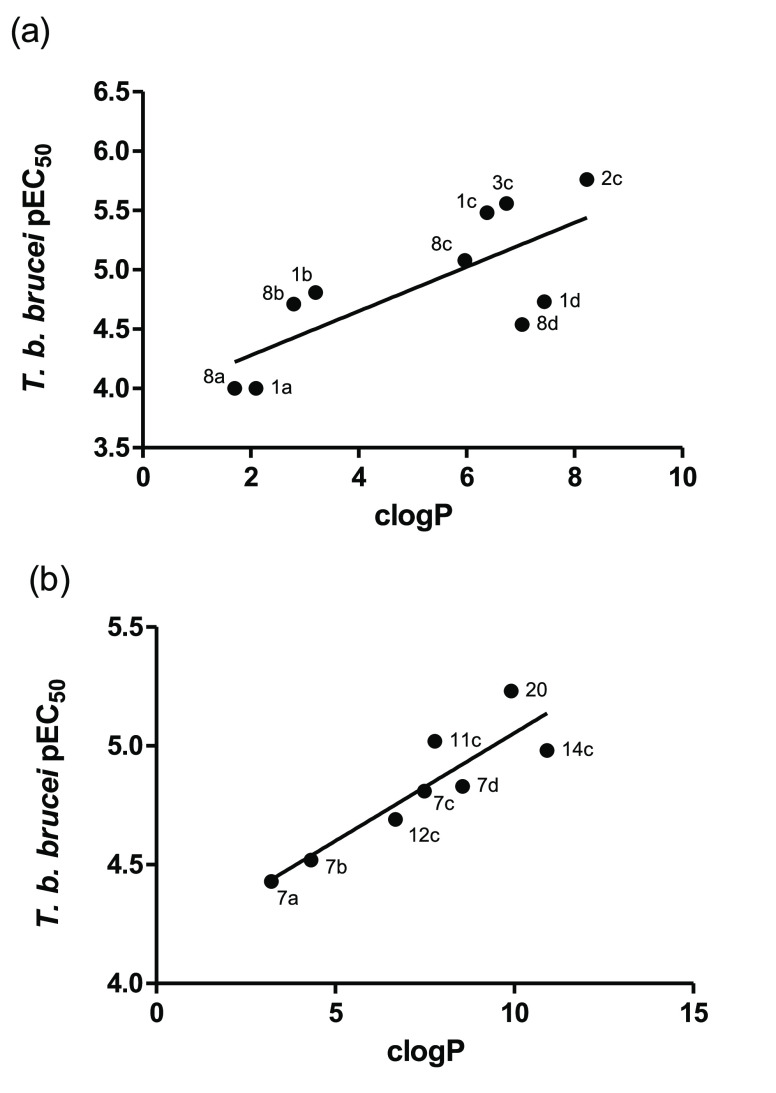
Correlations of cLogP versus *T. b. brucei* pIC_50_ growth inhibition for (a) cationic and (b) noncationic derivatives.

A positive correlation between clogP and the cellular activity against *T. brucei* was observed for the cationic derivatives **1a**–**d**, **2c**, **3c**, and **8a**–**d** (Figure 1a) and the noncationic derivatives **7a**–**d**, **11c**, **12c**, **14c**, and **20** (Figure 1b), although this was disconnected from inhibition of purified rTAO. A similar trend was observed by West and co-workers in a series of noncationic TAO inhibitors structurally related to ascofuranone. However, in this case, clogP also correlated with TAO inhibition.^23^ The positive effect of compound lipophilicity on the efficacy against *T. brucei* of the derivatives possibly reflects an increase in the permeability of the compounds through the cell and/or mitochondrial membranes, in agreement with previous studies on mitochondrion-targeted antiparasitic compounds.^8−10^ As reported previously, the accumulation of cationic compounds (e.g., **1a**–**d**, **2c**, **3c**, and **8a**–**d**) in the *T. brucei* mitochondrion is expected to affect the mitochondrial membrane potential Ψ_m_ by disruption of mitochondrial functions involved in maintaining the ion gradients.^11,25^ Hence, the absence of correlation between rTAO inhibition and *T. brucei* growth is probably the result of several factors including activity against multiple targets.

To conclude, this study showed that the replacement of TPP^+^ or quinolin-1-ium groups with imidazoline- and benzamidine-based mitochondrion-targeting cations was detrimental to the enzymatic and cellular activity of TAO inhibitors compared with previous series having the same 2,4-dihydroxy-6-methylbenzoic acid head. The comparatively weak micromolar range activity against TAO of these compounds illustrates the difficulty of modulating the tail region of TAO inhibitors with polar substituents without losing efficacy. Nevertheless, the 2-(phenylamino)imidazolin-3-ium group (**3c**) provided an inhibitor that was active against TAO and *T. brucei* in the low micromolar range, with adequate selectivity versus mammalian HEK cells.

## Experimental Section

### *T. brucei* Susceptibility Assays

BSF trypanosomes of monomorphic strains Lister 427 (WT), multi-drug-resistant clone B48, and the AQP1-3 KO, which lacks all aquaglyceroporins,^26^ were grown in complete HMI-9 with 10% fetal bovine serum, exactly as described, and tested using a standard resazurin-based assay with 23 doubling dilutions for each compound starting at 100 μM.^27^ Human embryonic kidney (HEK) cells were cultured and assayed with a resazurin-based assay exactly as described previously.^9^ EC_50_ values were calculated by nonlinear regression with an equation for a sigmoid curve with variable slope (Prism 8.0, GraphPad).

### Inhibition of rTAO

The test compounds were assayed as inhibitors of the ubiquinol oxidase activity of purified ΔMTS-TAO by recording the absorbance change of ubiquin-1-ol at 278 nm exactly as previously described.^9^ Briefly, determination of ΔMTS-TAO activity was performed on a V-630 Jasco UV–vis spectrophotometer (Jasco Corporation, Tokyo, Japan) by measuring the change in absorbance of the substrate ubiquinol (ε_278_ = 15,000 M^–1^ cm^–1^) at 278 nm over a period of 2 min in a 1 cm cuvette. The recombinant enzyme was preincubated for 2 min in a 50 mM Tris-HCl (pH 7.4) buffer containing the detergent octaethylene glycol monododecylether (0.05% (w/v)) in a total reaction volume of 1 mL at 25 °C. Reactions were initiated by the addition of ubiquinol to the cuvette. The inhibition reaction assay was performed by preincubating a fixed amount of rTAO with varying amounts of the inhibitor for 2 min in the same buffer before adding the substrate. Ascofuranone was used as positive control whereas DMSO was used as negative control. Control experiments were also carried out throughout the experiment to verify that there was no autoxidation of ubiquinol in the medium. Residual activities were plotted against the corresponding inhibitor concentration to generate the IC_50_ value using GraphPad Prism.

## Supporting Information Available

Synthesis and NMR spectra of all the target compounds **1a**–**d**, **2c**, **3c**, **20**, and **21** (PDF)

## Abbreviations

BSF trypanosome: bloodstream form trypanosome
DIAD: diisopropylazodicarboxylate
DMAP: 4-dimethylaminopyridine
HAT: human African trypanosomiasis
MTS: mitochondrion-targeting sequence
NADPH: nicotinamide adenine dinucleotide phosphate
PyBOP: benzotriazol-1-yloxytripyrrolidinophosphonium hexafluorophosphate
RF: resistance factor
TAO: trypanosome alternative oxidase
TPP: triphenylphosphonium
WT: wild-type
