# Supplementary material for: Imidazoline- and Benzamidine-Based Trypanosome Alternative Oxidase Inhibitors: Synthesis and Structure–Activity Relationship Studies
Source: ACS Med Chem Lett. 2022 Jan 28;13(2):312–8. doi: 10.1021/acsmedchemlett.1c00717 (PMC8842630; doi:10.1021/acsmedchemlett.1c00717)

Electronic Supporting Information

Imidazoline- and benzamidine-based trypanosome alternative oxidase inhibitors: synthesis and SAR studies

David Cisneros[a], Eduardo J. Cueto–Díaz[a†], Tania Medina–Gil[a], Rebecca Chevillard[a], Teresa Bernal–Fraile[a], Ramón López–Sastre[a], Mustafa M. Aldfer[c], Marzuq A. Ungogo[c], Hamza A. A. Elati, Natsumi Arai[b], Momoka Otani[b], Shun Matsushiro[b], Chiaki Kojima[b], Godwin U. Ebiloma[b,d], Tomoo Shiba[b], Harry P. de Koning[c], Christophe Dardonville[a*]

[a] Instituto de Química Médica, IQM–CSIC, Juan de la Cierva 3, E–28006 Madrid, Spain.

[b] Graduate School of Science and Technology, Department of Applied Biology, Kyoto Institute of Technology, Kyoto 606-8585, Japan.

[c] Institute of Infection, Immunity and Inflammation, College of Medical, Veterinary and Life Sciences, University of Glasgow, Glasgow, United Kingdom.

[d] School of Health and Life Sciences, Teesside University, Middlesbrough TS1 3BX, UK

Table of Contents Page

1) Synthesis of **1a**–**d** S3
2) Synthesis of **2c** and **3c** S14
3) Synthesis of **19** and **20** S18
4) NMR spectra of **1a**–**d**, **2c**, **3c**, **8a**–**d** S21

Experimental part

Chemistry. Anhydrous solvents were purchased from Fluka (DMSO) and Acros Organics (DMF, THF and CH_3CN). Monitoring of the reactions was carried out by thin layer chromatography (TLC) on silica-coated aluminum plates (ϕ 0.2 mm; Merck 60 F254) revealed under 254 and 365 nm UV lamp or using PMA (phosphomolybdic acid; 5% w/v in ethanol) or ninhydrin. For preparative TLC, UNIPLATE™ preparative silica plates (20×20 cm; 2000 microns) were used. Flash chromatography was performed with silica gel (Merck, 0.040-0.063 nm) in glass column or with Isolute SI prepacked cartridges using a FlashMaster Personal apparatus coupled to a Gilson UV/VIS-151 detector and FC203B fraction collector. HPLC–MS was performed with a Waters Alliance 2695 HPLC module integrated with a diode-array detector (PDA 2996), coupled to a quadrupole mass spectrometer (Micromass ZQ) with electrospray ionization mode (ESI). Three different cone voltages 20, 40 and 60 ēV were used. Analytical HPLC was performed with a SunFire C18-3.5 µm column (2.1 mm × 50 mm) detecting between 192 and 700 nm. The mobile phase of chromatography was acetonitrile (A) and MilliQ ultra-pure water (B) with 0.5% formic acid. ^{1}H- and ^{13}C-NMR spectra were recorded on a Bruker AVANCE-300, Varian Inova-400, Varian-Mercury-400, and Varian-500. Chemical shifts (δ) were referenced to $CDCl_3$ (δ_H = 7.26 ppm; δ_C = 77.16 ppm), DMSO-*d*$_6$ (δ_H = 2.5 ppm; δ_C = 39.52 ppm), and CD_3OD (δ_H = 3.31 ppm; δ_C = 49.0 ppm). Melting points were measured with the Mettler Toledo MP270 melting point apparatus.

1) Synthesis of benzamidine derivatives **1a–c**

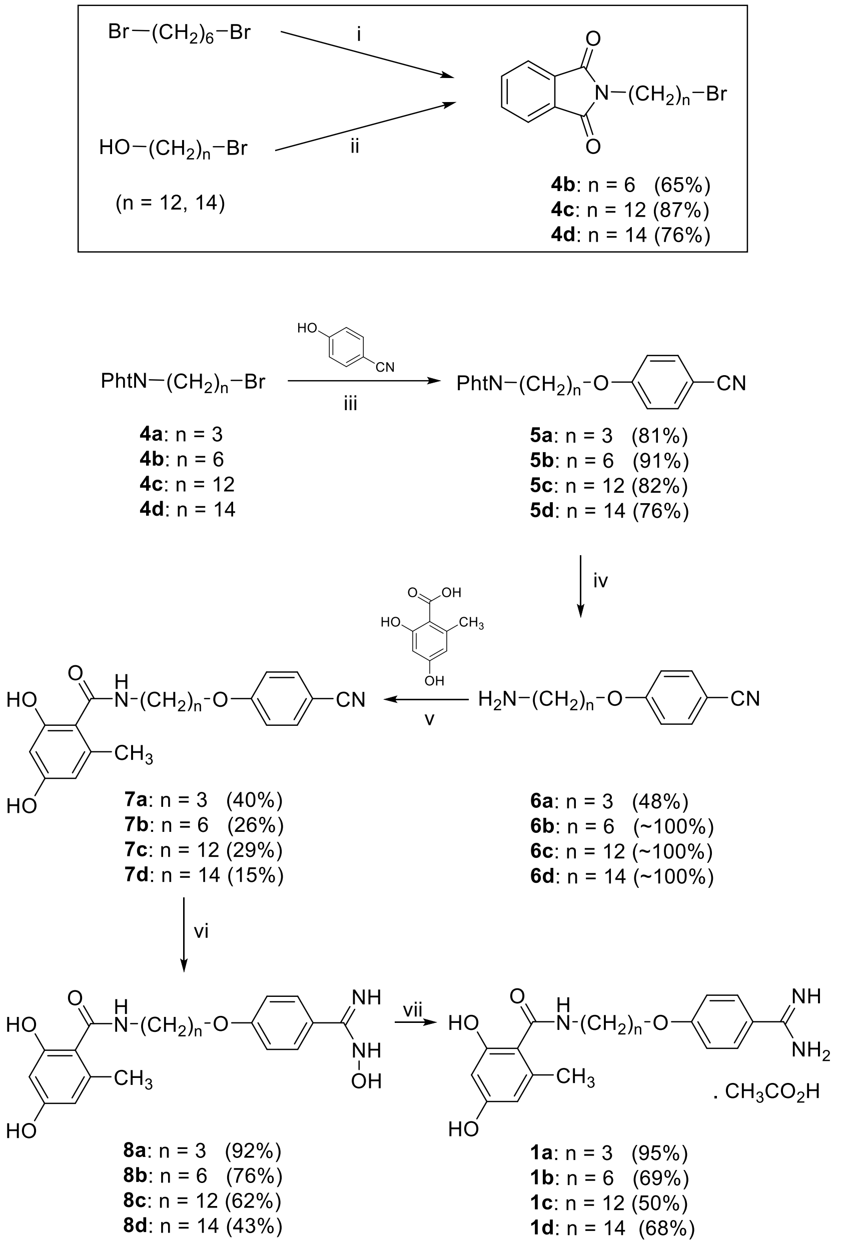

[a]Reagents and conditions. (i) $PhtN^{-}K^{+}$, DMF, rt, 20 h; (ii) PhtNH, Ph_3P, DIAD, THF, 0 ºC then rt, 20 h. (iii) K_2CO_3, CH_3CN, 80 ºC, 24 h; (iv) $N_2H_4{\cdot}H_2O$, EtOH, 80 ºC, 12 h; (v) For **7a**, **7c**, and **7d**: EDC·HCl, DMAP, CH_3CN, 80 ºC, 20 h. For **7b**: PyBOP, DIPEA, DMF, rt, 18 h; (vi) $NH_2OH{\cdot}HCl$, ^{t}BuOK, DMSO, rt, 4 days; (vii) 1) Ac_2O, AcOH, 15 min, 2) H_2, 5% Pd-C, AcOH, rt, 12 h.

***N*-(3-(4-carbamimidoylphenoxy)propyl)-2,4-dihydroxy-6-methylbenzamide acetate salt (1a)**. Acetic anhydride (15.6 µL, 0.16 mmol) was added to a stirred solution of **7a** (19.6 mg, 0.055 mmol) in acetic acid (1 mL). The reaction mixture was stirred at room temperature for 15 min. Then, 5% Pd/C (19 mg) was added and the reaction mixture was

hydrogenated (H_2 balloon) overnight at room temperature. Acetic acid (1 mL) was added and the crude reaction mixture was filtered on Celite. The filter cake was rinsed successively with AcOH (3 mL) and MeOH (3 mL), and the filtrate was evaporated under vacuum to yield a greyish solid. Recrystallization from MeOH/Et_2O at 4 ºC yielded benzamidine **1a** as acetate salt. Purplish solid (21 mg, 95%). HPLC–MS (UV)> 95%. ^{1}H NMR (400 MHz, DMSO-d_6) δ 11-9.0 (br, 6H), 8.36 (br, 1H), 7.78 (d, J = 8.5 Hz, 2H), 7.09 (d, J = 8.5 Hz, 2H), 6.12 (d, J = 2.2 Hz, 1H), 5.99 (d, J = 2.2 Hz, 1H), 4.15 (t, J = 6.5 Hz, 2H), 3.34 (t, J = 6.7 Hz, 2H), 2.14 (s, 3H), 1.94 (p, J = 6.7 Hz, 2H), 1.68 (s, 3H). ^{13}C NMR (101 MHz, DMSO-d_6) δ 175.9, 168.2, 165.1, 162.4, 158.3, 157.5, 137.3, 129.5, 121.2, 116.0, 114.6, 107.7, 100.4, 66.0, 35.5, 28.8, 24.9, 20.1. LRMS (ESI$^+$) m/z 344 $(M+H)^+$. HRMS (ESI$^+$) m/z 343.1532 ($C_{18}H_{21}N_3O_4$ requires 343.1532).

***N*-(6-(4-carbamimidoylphenoxy)hexyl)-2,4-dihydroxy-6-methylbenzamide acetate salt (1b).** Acetic anhydride (14 µL, 0.15 mmol) was added to a stirred solution of **7b** (20 mg, 0.05 mmol) in glacial AcOH (1 mL). The mixture was stirred at room temperature for 15 min, then Pd-C 5% (20 mg) was added to the flask. The reaction mixture was hydrogenated at atmospheric pressure and room temperature for 19 h. The mixture was diluted with AcOH (1 mL) and filtered on Celite. The filter cake was rinsed with AcOH (3 mL) and MeOH (3 mL). The filtrate was evaporated under vacuum to give crude **1b** as brownish oil. The crude compound was dissolved in EtOH and Et_2O was added to precipitate the product. The flask was allowed to stand at 4 ºC for a few days and the precipitate was collected and rinsed with Et_2O to give **1b** as greyish solid (15.3 mg, 69%). HPLC–MS (UV) > 95%. ^{1}H NMR (500 MHz, DMSO-d_6) δ 9.98 (br, 6H), 7.88 (brs, 1H), 7.78 (d, J = 8.4 Hz, 2H), 7.11 (d, J = 8.4 Hz, 2H), 6.12 (d, J = 2.3 Hz, 1H), 6.03 (d, J = 2.3 Hz, 1H), 4.07 (t, J = 6.5 Hz, 2H), 3.17 (t, J = 7.0 Hz, 2H), 2.12 (s, 3H), 1.78-1.68 (m, 5H), 1.54 – 1.33 (m, 6H). ^{13}C NMR (126 MHz, DMSO-d_6) δ 175.6, 167.8, 165.2, 162.6, 158.2, 156.4, 137.0, 129.6, 120.6, 116.5, 114.7, 108.0, 100.1, 68.0, 38.6, 29.0, 28.5, 26.1, 25.1, 24.2, 19.8. HRMS (ESI$^+$) m/z 385.2003 ($C_{21}H_{27}N_3O_4$ requires 385.2002).

***N*-(12-(4-carbamimidoylphenoxy)dodecyl)-2,4-dihydroxy-6-methylbenzamide acetate salt (1c).** Acetic anhydride (14 µL, 0.15 mmol) was added to a stirred solution of **7c** (24.5 mg, 0.05 mmol) in glacial AcOH (1 mL). The mixture was stirred at room temperature for 15 min, then Pd-C 5% (20 mg) was added to the flask. The reaction mixture was hydrogenated at atmospheric pressure and room temperature for 15 h. The mixture was diluted with AcOH (1 mL) and filtered on Celite. The filter cake was rinsed

successively with AcOH (3 mL) and MeOH (3 mL). The filtrate was evaporated under vacuum to give crude **1c** as brownish oil (21 mg). Recrystallization from MeOH/Et_2O at 4 ºC yielded a precipitate which was collected and rinsed with Et_2O to give **1c** as purplish solid (13.4 mg, 50%). HPLC–MS (UV) ≥ 95%. ^{1}H NMR (300 MHz, DMSO-d_6) δ 10.94-8.96 (br, 3H), 7.97 (br, 1H), 7.77 (d, *J* = 8.5 Hz, 2H), 7.10 (d, *J* = 8.5 Hz, 2H), 6.10 (d, *J* = 2.2 Hz, 1H), 6.01 (d, *J* = 2.2 Hz, 1H), 4.05 (t, *J* = 6.5 Hz, 2H), 3.14 (t, *J* = 6.9 Hz, 2H), 2.13 (s, 3H), 1.81-1.60 (m, 5H), 1.50 – 1.22 (m, 18H). ^{13}C NMR (101 MHz, DMSO-d_6) δ 176.6, 168.3, 165. 6, 163.0, 158.7, 157.3, 137.6, 130.0, 121.3, 116.6, 115.1, 114.5, 108.4, 100.7, 68.4, 39.2, 29.6, 29.5, 29.4, 29.23, 29.19, 28.9, 26.9, 25.9, 25.2, 20.4. LRMS (ESI^+) *m/z* 470 $(M+H)^+$.

***N*-(14-(4-carbamimidoylphenoxy)tetradecyl)-2,4-dihydroxy-6-methylbenzamide acetate salt (1d).** Acetic anhydride (8 µL, 0.08 mmol) was added to a stirred solution of **7d** (14 mg, 0.027 mmol) in glacial AcOH (1 mL). The mixture was stirred at room temperature for 15 min, then Pd-C 5% (15 mg) was added to the flask. The reaction mixture was hydrogenated at atmospheric pressure and room temperature for 6 h. The mixture was diluted with AcOH (3 mL) and filtered on Celite. The filter cake was rinsed successively with AcOH (2 mL) and MeOH (3 mL). The filtrate was evaporated under vacuum to give crude **1d** as oil (19 mg). The product was purified by two successive preparative thin-layer chromatography eluting with CH_2Cl_2/MeOH: 2/1 and $CHCl_3$/MeOH: 3/1. Compound **1d** was obtained as purplish amorphous solid (10.2 mg, 68%). HPLC–MS (UV) > 95%. ^{1}H NMR (400 MHz, DMSO-d_6) δ 10.3-9.2 (br, 5H), 7.86 (brs, 1H), 7.78 (d, *J* = 8.8 Hz, 2H), 7.11 (d, *J* = 8.8 Hz, 2H), 6.11 (d, *J* = 2.3 Hz, 1H), 6.03 (d, *J* = 2.3 Hz, 1H), 4.06 (t, *J* = 6.5 Hz, 2H), 3.14 (t, *J* = 6.9 Hz, 2H), 2.12 (s, 3H), 1.79 – 1.63 (m, 3H), 1.53 – 1.16 (m, 22H). ^{13}C NMR (101 MHz, DMSO-d_6) δ 175.6, 167.7, 167.2, 164.9, 162.6, 158.1, 156.4, 137.0, 129.6, 120.8, 116.5, 114.6, 108.0, 100.1, 68.0, 30.4, 29.1, 29.0, 28.8, 28.7, 28.5, 26.4, 25.4, 24.3, 19.8.

***N*-(6-bromohexyl)phthalimide (4b).** A mixture of potassium phthalimide (5 g, 26.9 mmol) and 1,6-dibromohexane (14.5 mL, 94 mmol) in dry DMF (50 mL) was stirred 20 h at room temperature. The solvent was removed under vacuum and the crude product was partitioned between water and EtOAc. The organic phase was dried ($MgSO_4$) and the solvent was evaporated. The product was purified by silica chromatography with hexane/EtOAc: 100/0 →90/10 to yield **4b** as colorless solid (5.7 g, 65%). ^{1}H NMR (400 MHz, DMSO-d_6) δ 7.90 – 7.78 (m, 4H), 3.56 (t, *J* = 7.1 Hz, 2H), 3.50 (t, *J* = 6.7 Hz, 2H),

1.77 (p, J = 6.9 Hz, 2H), 1.59 (p, J = 7.3 Hz, 2H), 1.46 – 1.34 (m, 2H), 1.34– 1.20 (m, 2H).

***N*-(12-bromododecyl)phthalimide (4c)**. To a stirred solution of 12-bromododecan-1-ol (1.824 g, 6.9 mmol), phthalimide (1.119 g, 7.6 mmol) and triphenylphosphine (1.995 g, 7.6 mmol) in anhydrous THF (60 mL) cooled with an ice-bath was added dropwise diisopropyldiazadicarboxylate (1.5 mL, 7.6 mmol). The reaction mixture was stirred at 0 ºC for 1 h and at room temperature for 21 h. The solvent was removed under vacuum and the crude oil was purified by silica chromatography with hexane/EtOAc: 100/0→90/10. Colorless solid (2.35 g, 87%). ^{1}H NMR (300 MHz, $CDCl_3$) δ 7.82 (dd, J = 5.4, 3.1 Hz, 2H), 7.69 (dd, J = 5.5, 3.1 Hz, 2H), 3.71 – 3.60 (m, 2H), 3.38 (t, J = 6.9 Hz, 2H), 1.83 (p, J = 7.0 Hz, 2H), 1.64 (p, J = 6.9 Hz, 2H), 1.41 – 1.20 (m, 16H). ^{13}C NMR (75 MHz, $CDCl_3$) δ 168.6, 134.0, 132.3, 123.3, 38.2, 34.2, 33.0, 29.60, 29.56, 29.52, 29.3, 28.9, 28.7, 28.3, 27.0.

***N*-(14-bromotetradecyl)phthalimide (4d).** To a stirred solution of 14-bromotetradecan-1-ol (226 mg, 0.77 mmol), phthalimide (125 mg, 0.85 mmol) and triphenylphosphine (223 mg, 0.85 mmol) in anhydrous THF (8 mL) cooled with an ice-bath was added dropwise diisopropyldiazadicarboxylate (0.167 mL, 0.85 mmol) under argon atmosphere. The reaction mixture was stirred at 0 ºC for 20 min and at room temperature for 22 h. The solvent was removed under vacuum and the crude oil was purified by silica chromatography with hexane/EtOAc: 100/0→90/10 to yield **4d** as yellowish oil that solidified upon standing at room temperature. Off-white glassy solid (246 mg, 76%). ^{1}H NMR (300 MHz, $CDCl_3$) δ 7.84 (dd, J = 5.4, 3.1 Hz, 2H), 7.70 (dd, J = 5.5, 3.1 Hz, 2H), 3.72 – 3.62 (m, 2H), 3.40 (t, J = 6.9 Hz, 2H), 1.85 (p, J = 6.9 Hz, 2H), 1.73 – 1.59 (m, 2H), 1.50 – 1.11 (m, 20H). ^{13}C NMR (75 MHz, $CDCl_3$) δ 168.6, 134.0, 132.3, 123.3, 38.2, 34.2, 33.0, 29.72, 29.68, 29.62, 29.58, 29.3, 28.9, 28.8, 28.3, 27.0.

4-(3-(1,3-dioxoisoindolin-2-yl)propoxy)benzonitrile (5a). To a solution of 4-cyanophenol (2.051 g, 17.2 mmol, 1 eq) in anhydrous CH_3CN (30 mL) was added *N*-(3-bromopropyl)-phthalimide (4.966 g, 18.5 mmol, 1.08 eq) and K_2CO_3 (2.615 g, 18.9 mmol, 1.1 eq). The reaction mixture was refluxed (80 °C) for 22 h under argon atmosphere. The reaction mixture was cooled with an ice-bath and the precipitate was filtered off. The filtrate was evaporated under vacuum to yield **5a** as colorless solid (4.264 g, 81%). HPLC–MS (UV)> 90%. M.p. 109.9 ºC. ^{1}H NMR (300 MHz, DMSO-d_6) δ 7.90-7.81 (m, 4H), 7.72 (dd, J = 8.8, 1.4 Hz, 2H), 6.96 (dd, J = 8.8, 1.5 Hz, 2H), 4.11 (t, J =

5.8 Hz, 2H), 3.76 (td, J = 6.7, 1.2 Hz, 2H), 2.08 (p, J = 6.2 Hz, 2H). ^{13}C NMR (75 MHz, $CDCl_3$) δ 168.5, 162.1, 134.2, 134.1, 132.2, 123.4, 119.3, 115.2, 104.2, 66.1, 35.3, 28.2.

4-((6-(1,3-dioxoisoindolin-2-yl)hexyl)oxy)benzonitrile (5b). A mixture of **4b** (939 mg, 3 mmol), 4-cyanophenol (378 mg, 3.2 mmol), and anhydrous K_2CO_3 (418 mg, 3 mmol) in dry CH_3CN (8 mL) was stirred at 80 ºC under argon atmosphere for 65 h. The precipitate was filtered off and rinsed successively with CH_3CN and CH_2Cl_2. The filtrate was evaporated under vacuum and the crude product was recrystallized from EtOAc (in the fridge overnight) and rinsed with hexane to yield **5b** as colorless solid (954 mg, 91%). ^{1}H NMR (300 MHz, $CDCl_3$) δ 7.83 – 7.71 (m, 2H), 7.71 – 7.59 (m, 2H), 7.54 – 7.44 (m, 2H), 6.90 – 6.79 (m, 2H), 3.92 (t, J = 6.4 Hz, 2H), 3.63 (t, J = 7.2 Hz, 2H), 1.81 – 1.68 (m, 2H), 1.72 – 1.57 (m, 2H), 1.48 – 1.32 (m, 2H).

4-((12-(1,3-dioxoisoindol-2-yl)dodecyl)oxy)benzonitrile (5c). A mixture of **4c** (1.31 g, 3.3 mmol), 4-cyanophenol (377 mg, 3.16 mmol), and anhydrous K_2CO_3 (458 mg, 3.3 mmol) in dry CH_3CN (20 mL) was stirred at 80 ºC under argon atmosphere for 28 h. The solvent was removed under vacuum and the crude solid was dissolved in CH_2Cl_2 and filtered on a fritted plate. The filter cake was rinsed with CH_2Cl_2 and the filtrate was evaporated under vacuum to give a colorless solid. Recrystallization from CH_3CN gave **5c** as colorless solid (1.154 g, 82%). M.p. 83.4 ºC. ^{1}H NMR (300 MHz, $CDCl_3$) δ 7.77 (dd, J = 5.5, 3.1 Hz, 2H), 7.64 (dd, J = 5.5, 3.1 Hz, 2H), 7.50 (d, J = 8.9 Hz, 2H), 6.86 (d, J = 8.9 Hz, 2H), 3.92 (t, J = 6.5 Hz, 2H), 3.60 (t, J = 7.3 Hz, 2H), 1.71 (dt, J = 8.1, 6.5 Hz, 2H), 1.60 (t, J = 7.3 Hz, 2H), 1.37 (h, J = 6.6 Hz, 2H), 1.30 – 1.17 (m, 14H). ^{13}C NMR (75 MHz, $CDCl_3$) δ 162.5, 159.2, 134.0, 132.1, 131.4, 129.3, 128.7, 128.5, 125.7, 119.4, 115.2, 103.7, 68.5, 41.2, 31.5, 30.7, 29.6, 29.43, 29.39, 29.1, 27.2, 26.9, 26.0.

4-((14-(1,3-Dioxoisoindolin-2-yl)tetradecyl)oxy)benzonitrile (5d). A mixture of **4d** (259 mg, 0.61 mmol), 4-cyanophenol (73 mg, 0.61 mmol), and anhydrous K_2CO_3 (89 mg, 0.61 mmol) in dry CH_3CN (3 mL) was stirred at 80 ºC under argon atmosphere for 24 h. The reaction mixture was allowed to cool to room temperature and the precipitate was collected by filtration on a fritted plate and rinsed with a small quantity of cold CH_3CN. The solid was dissolved in CH_2Cl_2 and filtered to eliminate the insoluble inorganic salts. The filtrate was evaporated under vacuum to yield **5d** as colorless solid. Recrystallization from CH_3CN gave **5d** as off-white solid (215 mg, 76%). ^{1}H NMR (300 MHz, $CDCl_3$) δ 7.84 (dd, J = 5.5, 3.0 Hz, 2H), 7.70 (dd, J = 5.4, 3.0 Hz, 2H), 7.59 – 7.53 (m, 2H), 6.96 – 6.88 (m, 2H), 3.99 (t, J = 6.5 Hz, 2H), 3.73 – 3.61 (m, 2H), 1.86 – 1.74 (m, 2H), 1.65 (q,

J = 7.2 Hz, 2H), 1.51 – 1.24 (m, 20H). ^{13}C NMR (75 MHz, $CDCl_3$) δ 168.6, 162.6, 134.1, 134.0, 132.3, 123.3, 119.5, 115.3, 103.8, 68.6, 38.2, 29.72, 29.68, 29.65, 29.61, 29.4, 29.3, 29.1, 28.7, 27.0, 26.1.

4-(3-aminopropoxy)benzonitrile (6a). A 250 mL round-bottomed flask was charged with **5a** (4.0 g, 13.1 mmol, 1 eq) in absolute ethanol (180 mL). Hydrazine monohydrate (4.2 mL, 86.4 mmol, 6.5 eq) was added to the suspension and the reaction mixture was refluxed until completion of the reaction (2 days) as shown by TLC (hexane/AcOEt: 1/1). The solvent was removed under vacuum and the crude product suspended in CH_2Cl_2 was filtered through Celite. The filtrate was evaporated to yield **6a** as orangish oil (1.1 g, 48%).^{1}H NMR (300 MHz, $CDCl_3$) δ 7.61 – 7.55 (m, 2H), 6.99 – 6.90 (m, 2H), 4.11 (t, J = 6.1 Hz, 2H), 2.92 (t, J = 6.7 Hz, 2H), 1.95 (p, J = 6.7 Hz, 2H).

4-((6-aminohexyl)oxy)benzonitrile (6b). A mixture of **5b** (872 mg, 2.5 mmol) and hydrazine monohydrate (1.2 mL, 25 mmol) in EtOH (30 mL) was refluxed for 17 h. The reaction mixture was filtered hot on a fritted plate and the precipitate was rinsed with EtOH. The filtrate was evaporated under vacuum and the crude residue was dissolved in CH_2Cl_2 and filtered. The filtrate was evaporated under vacuum and the solid was dried under high-vacuum to yield **6b** as off-white paraffin-like solid (545 mg, quantitative). HPLC–MS (UV) = 90%. ^{1}H NMR (300 MHz, DMSO-d_6) δ 7.80 – 7.65 (m, 2H), 7.14 – 7.03 (m, 2H), 4.04 (t, J = 6.5 Hz, 2H), 2.56 – 2.51 (m, 2H), 2.83 – 2.37 (br, 2H), 1.79 – 1.64 (m, 2H), 1.47 – 1.25 (m, 6H).

4-((12-aminododecyl)oxy)benzonitrile (6c). A mixture of **5c** (1 g, 2.25 mmol) and hydrazine monohydrate (1.1 mL, 22.5 mmol) in EtOH (50 mL) was refluxed for 23 h. The cool reaction mixture was filtered on a fritted plate and the precipitate was rinsed with EtOH. The filtrate was evaporated under vacuum and the crude residue was dissolved in CH_2Cl_2. The insoluble solid was filtered off and the filtrate was evaporated under vacuum. The resulting solid was dried under high-vacuum to yield **6c** as colorless solid (680 mg, quantitative). HPLC–MS (UV) > 90%. ^{1}H NMR (300 MHz, $CDCl_3$) δ 7.50 (d, J = 8.8 Hz, 2H), 6.86 (d, J = 8.8 Hz, 2H), 3.92 (t, J = 6.5 Hz, 2H), 2.61 (t, J = 6.9 Hz, 2H), 1.73 (dq, J = 8.3, 6.5 Hz, 2H), 1.66 – 1.15 (m, 18H). ^{13}C NMR (75 MHz, $CDCl_3$) δ 168.5, 162.5, 134.0, 133.9, 132.2, 123.2, 115.3, 68.5, 38.1, 29.6, 29.54, 29.50, 29.35, 29.2, 29.0, 28.7, 26.9, 26.0. LRMS (ESI^{+}) m/z 303 $(M+H)^{+}$.

4-((14-Aminotetradecyl)oxy)benzonitrile (6d). A mixture of **5d** (363 mg, 0.79 mmol) and hydrazine monohydrate (0.4 mL, 7.9 mmol) in absolute EtOH (20 mL) was refluxed for 14 h. The reaction mixture was cooled with an ice-bath and the precipitate was filtered with a fritted plate and rinsed with EtOH. The filtrate was evaporated under vacuum to give a crude solid. The solid was dissolved in CH_2Cl_2 and the insoluble precipitate was filtered off. The filtrate was evaporated under vacuum to yield **6d** as off-white solid (261 mg, quantitative). ^{1}H NMR (300 MHz, CD_3OD) δ 7.93 – 7.80 (m, 2H), 7.23 (d, *J* = 8.5 Hz, 2H), 4.27 (t, *J* = 6.4 Hz, 2H), 3.03 (t, *J* = 7.4 Hz, 2H), 2.11 – 1.97 (m, 2H), 1.87 – 1.34 (m, 22H).

***N*-(3-(4-cyanophenoxy)propyl)-2,4-dihydroxy-6-methylbenzamide (7a).** An oven-dried round-bottomed flask was charged with orsellinic acid (299 mg, 1.78 mmol, 1.0 eq), EDC (407 mg, 2.12 mmol, 1.2 eq), and DMAP (46 mg, 0.38 mmol, 0.2 eq) in anhydrous CH_3CN (15 mL). The reaction mixture was stirred 30 minutes at room temperature and a solution of **6a** (465 mg, 2.13 mmol, 1.2 eq) in anhydrous CH_3CN (10 mL) was added dropwise at room temperature. The reaction mixture was refluxed overnight and the solvent was removed under vacuum. The crude product was partitioned between CH_2Cl_2 and HCl 0.1 M. The aqueous phase was extracted with CH_2Cl_2 and the combined organic extracts were washed with brine, dried ($MgSO_4$) and evaporated. The crude product was recrystallized from $CHCl_3$ to give **7a** as yellow solid (232 mg, 40%). HPLC–MS (UV)> 95%. ^{1}H NMR (300 MHz, $CDCl_3$+CD_3OD) δ 7.43 (d, J = 8.8 Hz, 2H), 6.82 (d, *J* = 8.8 Hz, 2H), 6.04 (s, 1H), 6.03 (s, 1H), 3.99 (t, *J* = 5.4 Hz, 2H), 3.44 (t, *J* = 6.6 Hz, 2H), 2.21 (s, 3H), 2.04 – 1.89 (m, 2H). ^{13}C NMR (75 MHz, CD_3OD) δ 170.2, 162.2, 159.1, 158.5, 138.4, 133.9, 119.1, 115.1, 112.5, 110.0, 103.4, 100.5, 66.1, 36.7, 28.6, 20.9. LRMS (ESI^+) *m/z* 327 $(M+H)^+$. HRMS (ESI^+) *m/z* 326.1273 ($C_{18}H_{18}N_2O_4$ requires 326.1267).

***N*-(6-(4-cyanophenoxy)hexyl)-2,4-dihydroxy-6-methylbenzamide (7b).** 4-((6-aminohexyl)oxy)benzonitrile **6b** (500 mg, 2.29 mmol) was dissolved in 10 mL of anhydrous DMF. Then, orsellinic acid (511 mg, 2.75 mmol), ((1*H*-benzo[*d*][1,2,3]triazol-1-yl)oxy)tri(pyrrolidin-1-yl)phosphonium hexafluorophosphate (PyBOP) (1.4 mg, 2.75 mmol) and *N,N* diisopropylethylamine (470 µl, 2.75 mmol) were successively added. The reaction was then kept under stirring at room temperature for 18 h. Thereafter, the solvent was evaporated and the crude solid was washed with brine (50 mL) and extracted with DCM (2×50 mL). The collected organic phase was dried over $MgSO_4$, filtered and concentrated under vacuum. The viscous solid crude was purified by flash

chromatography column (SiO_2; gradient from 0–50% AcOEt in Hexane) to afford **7b** as white powder (190 mg, 26%). HPLC–MS (UV) > 95%. ^{1}H NMR (300 MHz, CD_3OD) δ 7.63 (d, *J* = 8.8 Hz, 2H), 7.04 (d, *J* = 8.9 Hz, 2H), 6.15 (2×s, 2H), 4.06 (t, *J* = 6.4 Hz, 2H), 3.35 (t, *J* = 6.9 Hz, 2H), 2.23 (s, 3H), 1.80–1.84 (m, 2H), 1.62–1.65 (m, 2H), 1.49–1.53 (m, 4H). ^{13}C NMR (100 MHz, CD_3OD) δ 171.7, 164.1, 160.0, 157.6, 139.0, 135.1, 120.2, 117.3, 116.5, 109.6, 104.4, 101.1, 69.4, 40.6, 30.2, 30.0, 27.7, 26.7, 20.1. HMRS (ESI$^+$) *m/z* 368.1754 ($C_{21}H_{24}N_2O_4$ requires 368.1736).

***N*-(12-(4-cyanophenoxy)dodecyl)-2,4-dihydroxy-6-methylbenzamide (7c).** An oven-dried round-bottomed flask with a screw cap was charged with orsellinic acid (196 mg, 1.17 mmol, 1.0 eq) in anhydrous CH_3CN (15 mL). EDC hydrochloride (268 mg, 1.4 mmol), and DMAP (34 mg, 0.28 mmol) were added portion wise and the reaction mixture was stirred 30 minutes at room temperature under argon atmosphere. Then, a solution of **6c** (423 mg, 1.4 mmol) dissolved in hot anhydrous CH_3CN (15 mL) was added dropwise (at room temperature) to the mixture of orsellinic acid. The flask was screw-capped and the resulting reaction mixture was heated at 85 ºC for 27 h. The solvent was removed under vacuum and the crude product was partitioned between CH_2Cl_2 and water. The organic phase was washed with brine, dried ($MgSO_4$) and evaporated to give a reddish crude. Chromatography (10 g SI) with CH_2Cl_2/EtOAC: 0→15% yielded **7c** as colorless solid (153 mg, 29%). HPLC–MS (UV) > 95%. M.p. 81.9 ºC. ^{1}H NMR (300 MHz, $CDCl_3$) δ 11.86 (brs, 1H), 7.50 (d, *J* = 8.9 Hz, 2H), 6.86 (d, *J* = 8.9 Hz, 2H), 6.22 (d, *J* = 2.5 Hz, 1H), 6.13 (dd, *J* = 2.5, 0.8 Hz, 1H), 5.87 (brs, 1H), 5.33 (brs, 1H), 3.92 (t, *J* = 6.5 Hz, 2H), 3.38 (td, *J* = 7.2, 5.6 Hz, 2H), 2.42 (s, 3H), 1.83 – 1.65 (m, 2H), 1.47-1-12 (m, 18H). ^{13}C NMR (75 MHz, $CDCl_3$) δ 170.7, 162.9, 162.5, 159.8, 137.2, 133.9, 119.3, 115.2, 110.8, 109.7, 103.5, 101.8, 68.4, 39.9, 29.49 (br), 29.44, 29.29, 29.22, 29.0, 27.1, 25.9, 22.8. LRMS (ESI$^+$) *m/z* 453 (M+H)$^+$. HRMS (ESI$^+$) *m/z* 452.2688 ($C_{27}H_{36}N_2O_4$ requires 452.2675).

***N*-(14-(4-Cyanophenoxy)tetradecyl)-2,4-dihydroxy-6-methylbenzamide (7d).** An oven-dried round-bottomed flask with a screw cap was charged with orsellinic acid (112 mg, 0.67 mmol) dissolved in a mixture of anhydrous CH_3CN (15 mL) and CH_2Cl_2 (5 mL). EDC hydrochloride (153 mg, 0.8 mmol) and DMAP (15 mg, 0.13 mmol) were added portion wise and the reaction mixture was stirred 30 minutes at room temperature under argon atmosphere. Then, a solution of **6d** (265 mg, 0.8 mmol) dissolved in hot anhydrous CH_3CN (15 mL) was added dropwise (at room temperature) to the mixture of orsellinic

acid. The flask was screw-capped and the resulting reaction mixture was heated at 80 ºC for 3 days. The solvent was removed under vacuum and the crude product was partitioned between CH_2Cl_2 and water. The organic phase was washed with brine, dried ($MgSO_4$) and evaporated to give a reddish crude. Chromatography (5 g SI) with CH_2Cl_2/EtOAC: 0→15% yielded **7d** as off-white semi-solid (48 mg, 15%). HPLC–MS (UV)> 90%. ^{1}H NMR (300 MHz, $CDCl_3$) δ 11.88 (s, 1H), 7.50 (dd, *J* = 9.1, 0.6 Hz, 2H), 6.86 (d, *J* = 9.1 Hz, 2H), 6.21 (d, *J* = 2.6 Hz, 1H), 6.13 (d, *J* = 2.6 Hz, 1H), 5.85 (brs, 2H), 5.02 (brs, 1H), 3.92 (t, *J* = 6.6 Hz, 2H), 3.38 (td, *J* = 7.2, 5.6 Hz, 2H), 2.42 (s, 3H), 1.82 – 1.64 (m, 2H), 1.63 – 1.09 (m, 22H). ^{13}C NMR (75 MHz, $CDCl_3$) δ 170.8, 162.8, 162.7, 159.1, 137.4, 134.1, 119.5, 115.3, 110.9, 110.2, 103.6, 102.1, 68.6, 40.2, 29.72, 29.66, 29.62, 29.5, 29.42, 29.35, 29.1, 27.2, 26.0, 22.9. LRMS (ESI$^+$) *m/z* 482 $(M+H)^+$.

2,4-dihydroxy-*N*-(3-(4-(*N*-hydroxycarbamimidoyl)phenoxy)propyl)-6-methylbenzamide (8a). Potassium *tert*-butoxide (101 mg, 0.83 mmol) was added at once to a stirred solution of hydroxylamine hydrochloride (57 mg, 0.83 mmol) in anhydrous DMSO (1 mL). The mixture was stirred 35 minutes at room temperature under argon atmosphere. The resulting hydroxylamine solution was added dropwise (under argon) to a Kimax tube containing a stirred solution of nitrile **7a** (54 mg, 0.166 mmol) in anhydrous DMSO (0.5 mL). The reaction mixture was stirred at room temperature (64 h) until complete consumption of the starting material. The solvent was removed by lyophilization and the crude residue was dissolved in MeOH to precipitate KBr. The supernatant was collected and evaporated under vacuum. Ether-mediated precipitation of the product dissolved in MeOH (at 4 ºC overnight) yielded a solid that was rinsed with Et_2O and dried. Compound **8a** was obtained as beige solid (55 mg, 92%). HPLC–MS (UV)> 95%. ^{1}H NMR (300 MHz, DMSO-d_6) δ 9.44 (brs, 2H), 7.94 (brt, *J* = 5.7 Hz, 1H), 7.59 (d, J = 8.8 Hz, 2H), 6.91 (d, J = 8.8 Hz, 2H), 6.16 (d, *J* = 2.2 Hz, 1H), 6.06 (d, *J* = 2.2 Hz, 1H), 5.70 (s, 2H), 4.06 (t, *J* = 6.4 Hz, 2H), 3.43 – 3.34 (m, 2H), 2.11 (s, 3H), 1.99 – 1.86 (m, 2H). ^{13}C NMR (75 MHz, DMSO-d_6) δ 168.1, 159.2, 158.3, 150.6, 137.2, 126.7, 125.7, 116.0, 113.9, 107.7, 100.4, 65.5, 35.7, 28.9, 20.1. LRMS (ESI$^+$) *m/z* 360 $(M+H)^+$. HMRS (ESI$^+$) *m/z* 359.1496 ($C_{18}H_{21}N_3O_5$ requires 359.1481).

2,4-dihydroxy-*N*-(6-(4-(*N*-hydroxycarbamimidoyl)phenoxy)hexyl)-6-methylbenzamide (8b). Potassium *tert*-butoxide (108 mg, 0.88 mmol) was added at once to a stirred solution of hydroxylamine hydrochloride (62 mg, 0.88 mmol) in anhydrous DMSO (1 mL). The mixture was stirred 30 minutes at room temperature under argon

atmosphere. The resulting hydroxylamine solution was added dropwise (under argon) to a Kimax tube containing a stirred solution of nitrile **7b** (65 mg, 0.18 mmol) in anhydrous DMSO (0.5 mL). The reaction mixture was stirred at room temperature (4 days) until complete consumption of the starting material. Ice (2 mL approximately) was added to the mixture and the tube was shaken vigorously. The tube was allowed to stand at 4 ºC overnight to precipitate the product. The tube was centrifuged and the supernatant was discarded. The precipitate was rinsed successively with water and Et_2O, and dried under vacuum to give **8b** as colorless powder (55 mg, 76%). HPLC–MS (UV) > 95%. ^{1}H NMR (500 MHz, CD_3OD) δ 7.59 – 7.53 (m, 2H), 6.97 – 6.91 (m, 2H), 6.15 (s, 2H), 4.02 (t, *J* = 6.4 Hz, 2H), 3.35 (t, *J* = 7.0 Hz, 2H), 2.23 (s, 3H), 1.80 (dt, *J* = 8.2, 6.4 Hz, 2H), 1.68 – 1.61 (m, 2H), 1.59 – 1.46 (m, 4H). ^{13}C NMR (126 MHz, CD_3OD) δ 171.8, 162.3, 160.0, 157.6, 156.4, 139.0, 130.6, 128.9, 125.3, 117.4, 115.4, 109.6, 101.1, 69.0, 40.6, 30.3, 30.2, 27.7, 26.8, 20.1. HRMS (ESI$^+$) *m/z* 401.1962 ($C_{21}H_{27}N_3O_5$ requires 401.1951).

2,4-Dihydroxy-*N*-(12-(4-(*N*-hydroxycarbamimidoyl)phenoxy)dodecyl)-6-methylbenzamide (8c). Potassium *tert*-butoxide (120 mg, 1 mmol) was added portion wise to a stirred solution of hydroxylamine hydrochloride (70 mg, 1 mmol) in anhydrous DMSO (1 mL). The mixture was stirred 30 minutes at room temperature under argon atmosphere. The resulting hydroxylamine solution was added dropwise (under argon) to a Kimax tube containing a stirred solution of nitrile **7c** (91 mg, 0.2 mmol) in anhydrous DMSO (0.5 mL). The reaction mixture was stirred at room temperature (6 days) until complete consumption of the starting material. Ice (2 mL approximately) was added to the mixture and the tube was shaken vigorously. The tube was allowed to stand at 4 ºC overnight to precipitate the product. The tube was centrifuged and the supernatant was discarded. The precipitate was rinsed with water and dried under vacuum. The solid was dissolved in little MeOH and little Et_2O was added. The precipitate was filtered off and the mother liquor was evaporated to dryness to give a colorless residue. Et_2O was added and the residue was triturated with a spatula to yield pure **8c** as off-white powder which was dried under vacuum (60 mg, 62%). HPLC–MS (UV) > 95%. ^{1}H NMR (300 MHz, DMSO-d_6) δ 9.43 (brs, exchangeable, 3H), 7.80 (brs, exchangeable, 1H), 7.58 (d, *J* = 8.5 Hz, 2H), 6.90 (d, *J* = 8.5 Hz, 2H), 6.11 (d, *J* = 2.2 Hz, 1H), 6.04 (d, *J* = 2.2 Hz, 1H), 5.70 (s, 2H), 3.96 (t, *J* = 6.5 Hz, 2H), 3.14 (d, *J* = 8.0 Hz, 2H), 2.13 (s, 3H), 1.70 (p, *J* = 6.8 Hz, 2H), 1.55-1.23 (m, 18H). ^{13}C NMR (75 MHz, DMSO-d_6) δ 167.7, 159.2, 158.0, 156.2, 150.6, 137.0, 126.7, 125.6, 116.5, 113.9, 108.0, 100.0, 67.4, 29.09, 29.05, 29.0,

28.8, 28.7, 26.4, 25.5, 19.7. LRMS (ESI^+) *m/z* 486 $(M+H)^+$. HRMS (ESI^+) *m/z* 485.2887 ($C_{27}H_{39}N_3O_5$ requires 485.2890).

2,4-Dihydroxy-*N*-(14-(4-(*N*-hydroxycarbamimidoyl)phenoxy)tetradecyl)-6-methylbenzamide (8d). Potassium *tert*-butoxide (46 mg, 0.38 mmol) was added portion wise to a stirred solution of hydroxylamine hydrochloride (27 mg, 0.38 mmol) in anhydrous DMSO (1 mL). The mixture was stirred 30 minutes at room temperature under argon atmosphere. The resulting hydroxylamine solution was added dropwise (under argon) to a Kimax tube containing a stirred solution of nitrile **7d** (37 mg, 0.077 mmol) in anhydrous DMSO (0.5 mL). The reaction mixture was stirred at room temperature (7 days) until complete consumption of the starting material. Ice (2 mL approximately) was added to the mixture and the tube was shaken vigorously. The tube was centrifuged (20 min at 4000 rpm) and the supernatant was removed. Water was added to the tube to rinse the precipitate; the tube was centrifuged and the supernatant was discarded. This cycle of washing/centrifuge was repeated once and the resulting precipitate was dried under high vacuum to give a crude solid. Silica chromatography with EtOAc yielded **8d** as off-white powder (17 mg, 43%). HPLC–MS (UV)> 90%. ^1H NMR (400 MHz, CD_3OD) δ 7.56 (d, *J* = 8.8 Hz, 2H), 6.93 (d, *J* = 8.8 Hz, 2H), 6.17 (d, *J* = 2.3 Hz, 1H), 6.15 (d, *J* = 2.3 Hz, 1H), 4.00 (t, *J* = 6.5 Hz, 2H), 3.33 (d, *J* = 7.0 Hz, 2H), 2.25 (s, 3H), 1.76 (dt, *J* = 8.2, 6.5 Hz, 2H), 1.59 (p, *J* = 7.0 Hz, 2H), 1.52 – 1.28 (m, 20H). ^{13}C NMR (75 MHz, CD_3OD+CD_3CN) δ 171.4, 161.8, 160.0, 157.8, 155.3, 139.1, 128.6, 126.1, 117.1, 115.3, 109.7, 101.0, 69.1, 40.6, 30.65, 30.60, 30.38, 30.34, 30.31, 30.2, 28.0, 27.0, 20.2. LRMS (ESI^+) *m/z* 514 $(M+H)^+$.

2) Synthesis of imidazoline derivatives **2c** and **3c**

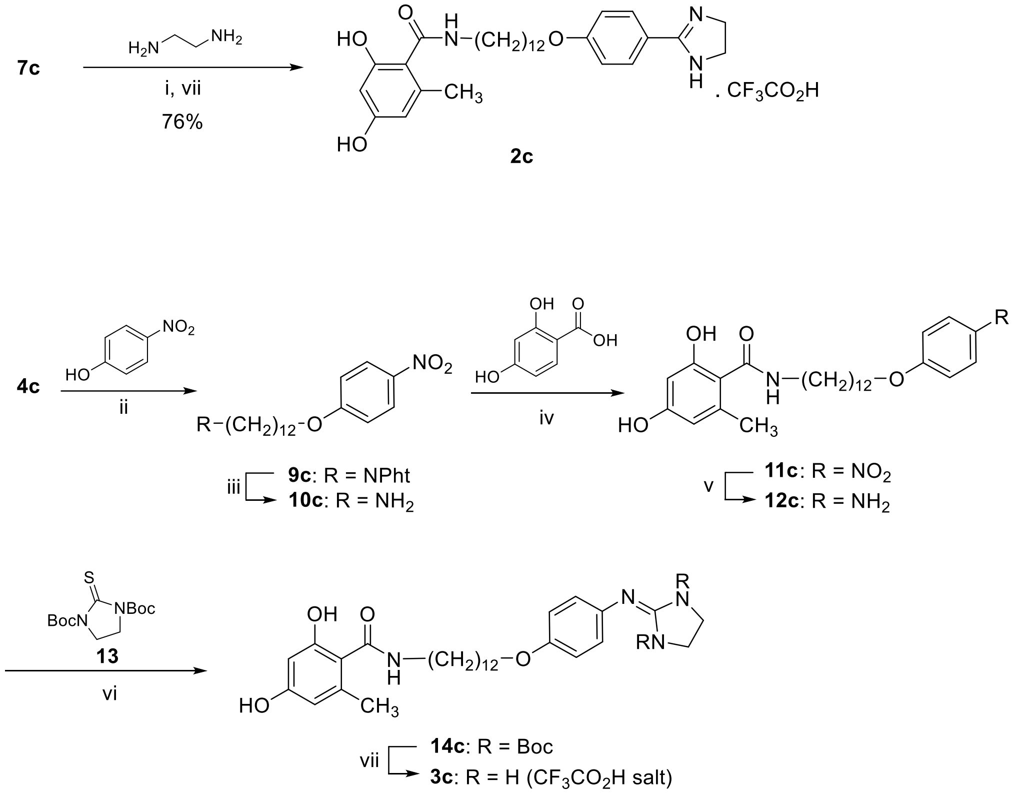

[a]Reagents and conditions: (i) P_2S_5, 1,2-ethylenediamine, sealed tube, 120 ºC, 2 h; (ii) K_2CO_3, CH_3CN, 80 ºC; (iii) $N_2H_4{\cdot}H_2O$, EtOH, 80 ºC; (iv) EDC·HCl, DMAP, CH_3CN, CH_2Cl_2, 80 ºC; (v) H_2, MeOH, Pd-C 5%; (vi) $HgCl_2$, Et_3N, DMF, 0 ºC to rt; (vii) CH_2Cl_2, TFA, 0 ºC.

2-(4-((12-(2,4-dihydroxy-6-methylbenzamido)dodecyl)oxy)phenyl)-4,5-dihydro-1H-imidazol-1-ium 2,2,2-trifluoroacetate (2c). Compound **7c** (50 mg, 0.11 mmol) and P_2S_5 (13.3 mg, 0.06 mmol) were dissolved in 1.5 mL of ethylenediamine in a (Kimax) sealed tube, and the tube was heated to 120 ºC for 2 h. The tube was cooled to room temperature, and 4 mL of iced-water were poured into the reaction mixture. Centrifugation yielded **2c** as free base (41.2 mg, 75%). The colourless solid was suspended in CH_2Cl_2 (1.8 mL). The flask was cooled with an ice-bath and TFA (1.8 mL) was added dropwise under argon atmosphere. The resulting mixture was stirred at 0 ºC for 2h. Finally, solvent was evaporated and a white solid was obtained. Precipitation with diethyl ether yielded the trifluoroacetate salt of **2c** (38.3 mg, 76%). HPLC–MS (UV) > 95%. M.p. 73.1–75.9 ºC. ^{1}H NMR (500 MHz, CD_3OD) δ 7.81 (d, J = 9.0 Hz, 2H), 7.13 (d, J = 9.0 Hz, 2H), 6.15 (s, 2H), 4.10 (t, J = 6.4 Hz, 2H), 4.05 (s, 4H), 3.33 (t, J = 7.0 Hz, 2H), 2.23 (s, 3H), 1.81

(m, 2H), 1.48 (p, 2H), 1.50–1.32 (m, 16 H). ^{13}C NMR (126 MHz, $CDCl_3$) δ 170.4, 165.9, 164.5, 159.6, 156.2, 137.6, 130.1, 116.0, 115.0, 113.7, 108.2, 99.7, 47.9, 44.3, 39.6, 36.8, 29.3, 29.18, 29.17, 28.98, 28.90, 28.6, 26.6, 25.6, 18.6. MS (ESI$^+$) *m/z* 497 [M+H]$^+$. HRMS (ESI$^+$) *m/z* 495.3099 ($C_{29}H_{41}N_3O_4$ requires 495.3097).

2-((4-((12-(2,4-dihydroxy-6-methylbenzamido)dodecyl)oxy)phenyl)amino)-4,5-dihydro-1*H* imidazol-1-ium 2,2,2-trifluoroacetate (3c). To a suspension of **14c** (42.5 mg, 0.06 mmol) in CH_2Cl_2 (1.8 mL) cooled with an ice-water bath was added dropwise TFA (5 mL) under argon atmosphere. The reaction mixture was stirred for 5 h at 0 ºC. Finally, the solvent was evaporated to give a colourless solid, which was triturated with Et_2O to yield **3c** (32.8 mg, 88%). HPLC–MS (UV) > 96%. M.p. 44.5–51.1 ºC. ^{1}H NMR (500 MHz, CD_3OD) δ 7.19 (d, *J* = 8.9 Hz, 2H), 6.99 (d, *J* = 8.9 Hz, 2H), 6.15 (s, 2H), 3.99 (t, *J* = 6.4 Hz, 2H), 3.74 (s, 4H), 3.33 (t, *J* = 7.3 Hz, 2H), 2.23 (s, 3H), 1.77 (m, 2H), 1.59 (p, *J* = 7.0 Hz, 2H), 1.50–1.31 (m, 16 H). ^{13}C NMR (126 MHz, CD_3OD) δ 170.4, 159.4, 158.7, 158.6, 156.2, 137.6, 127.6, 126.2, 116.0, 115.2, 108.2, 99.7, 67.9, 42.7, 39.3, 29.27, 29.24, 29.23, 29.00, 28.98, 28.86, 26.65, 25.68, 18.66. HRMS (ESI$^+$) *m/z* 510.3212 ($C_{29}H_{42}N_4O_4$ requires 510.3206).

2-(12-(4-nitrophenoxy)dodecyl)isoindoline-1,3-dione (9c). A mixture of **4c** (1.2 g, 3.04 mmol), 4-nitrophenol (423 mg, 3.04 mmol), and anhydrous K_2CO_3 (421 mg, 3.04 mmol) was stirred at 80 ºC under argon atmosphere for 21 h. The solvent was removed under vacuum and the crude solid was dissolved in CH_2Cl_2 and filtered on a fritted plate. The filter cake was rinsed with CH_2Cl_2 and the filtrate was evaporated under vacuum to give **9c** as yellow solid (1.35g, 98%). M.p. 97.0–99.0 ºC. ^{1}H NMR (400 MHz, $CDCl_3$) δ 8.18 (d, *J* = 9.2 Hz, 2H), 7.83 (dd, *J* = 5.5, 3.1 Hz, 2H), 7.70 (dd, *J* = 5.5, 3.0 Hz, 2H), 6.93 (d, *J* = 9.2 Hz, 2H), 4.04 (t, *J* = 6.5 Hz, 2H), 3.67 (t, *J* = 7.3 Hz, 2H), 1.80 (m, 2H), 1.66 (t, *J* = 7.3 Hz, 2H), 1.50-1.19 (m, 16H). ^{13}C NMR (101 MHz, $CDCl_3$) δ 168.5, 164.3, 161.3, 133.9, 132.2, 125.9, 123.1, 114.4, 68.9, 38.1, 29.50, 29.47, 29.44, 29.3, 29.2, 29.0, 28.6, 26.9, 25.9.

12-(4-nitrophenoxy)dodecan-1-amine (10c). A mixture **9c** (1.32 g, 2.92 mmol), and hydrazine monohydrate (1.42 mL, 29.2 mmol) in ethanol (50 mL) was refluxed for 19 h. The cool reaction mixture was filtered on a fritted plate and the precipitate was rinsed with ethanol. The filtrate was evaporated under vacuum and the crude residue was dissolved in CH_2Cl_2. The insoluble solid was filtered off and the filtrate was evaporated under vacuum. The resulting solid was dried under high-vacuum to yield **10c** as yellow

solid (939 mg, 100%). HPLC–MS (UV) > 95%. M.p. 63.6–67.4 ºC. ^{1}H NMR (400 MHz, $CDCl_3$) δ 8.17 (d, *J* = 9.2 Hz, 2H), 6.92 (d, *J* = 9.2 Hz, 2H), 4.03 (t, *J* = 6.5 Hz, 2H), 2.67 (t, *J* = 7.0 Hz, 2H), 1.79 (m, 2H), 1.56-1.17 (m, 18H). ^{13}C NMR (101 MHz, $CDCl_3$) δ 163.2, 140.3, 124.9, 113.4, 76.2, 67.9, 41.2, 32.7, 28.59, 28.56, 28.53, 28.50, 28.48, 28.28, 27.9, 25.9, 24.9. MS (ESI$^+$) *m*/*z* 324 $[M+H]^+$.

2,4-dihydroxy-6-methyl-*N*-(12-(4-nitrophenoxy)dodecyl)benzamide (11c). An oven-dried round-bottomed flask with screw-cap was charged with orsellinic acid (400 mg, 2.38 mmol) in anhydrous CH_3CN (18 mL). EDC hydrochloride (547 mg, 2.85 mmol) and DMAP (58 mg, 0,476 mmol) in anhydrous CH_3CN (18 mL) under argon atmosphere in an ice bath were added dropwise to the orsellinic acid suspension at room temperature. Then, a solution of **10c** dissolved in hot anhydrous CH_3CN (20 mL) was added dropwise to the reaction mixture. The flask was screw-capped and the resulting reaction mixture was heated at 85 ºC for 4 days. The solvent was removed under vacuum and the crude product was partitioned between CH_2Cl_2 and water. The organic phase was washed with brine, dried ($MgSO_4$) and evaporated to give a reddish crude. Chromatography (12g SI) with Hexane/EtOAC: 10→25% yielded **11c** as colourless solid (390 mg, 35%). HPLC–MS (UV) > 95%. M.p. 102.3–106.3 ºC. ^{1}H NMR (500 MHz, $CDCl_3$) δ 8.19 (d, *J* = 9.3 Hz, 2H), 6.93 (d, *J* = 9.3 Hz, 2H), 6.29 (d, *J* = 2.3 Hz, 1H), 6.20 (d, *J* = 2.3 Hz, 1H), 5.98 (s, 1H), 4.04 (t, *J* = 6.5 Hz, 2H), 3.45 (m, 2H), 2.47 (s, 3H), 1.81 (m, 2H), 1.61 (p, *J* = 7.4, 7.2 Hz, 2H), 1.49–1.27 (m, 16 H). ^{13}C NMR (126 MHz, $CDCl_3$) δ 170.6, 164.3, 162.9, 158.6, 141.3, 137.3, 125.9, 114.4, 110.6, 110.2, 102.0, 68.9, 40.0, 29.71, 29.51, 29.49, 29.40, 29.29, 29.21, 28.96, 27.0, 25.9, 22.8. MS (ESI$^-$) *m*/*z* 472 $[M-H]^-$. HRMS (ESI$^+$) *m/z* 472.2574 ($C_{26}H_{36}N_2O_6$ requires 472.2573).

***N*-(12-(4-aminophenoxy)dodecyl)-2,4-dihydroxy-6-methylbenzamide (12c).** 5% Pd/C (65.9 mg) was added to a cooled solution of **11c** (340 mg, 0.719 mmol) in methanol (20 mL), at room temperature in a 200 mL hydrogenation flask. The reduction was carried out at a pressure of 39 psi (2.7 bar) in a Parr hydrogenator for 3.5 h. Then, the suspension was filtered on celite. The solvent was evaporated under vacuum and **12c** was obtained as red powder (302 mg, 95%). HPLC–MS (UV) > 95%. M.p. 104.9–108.0 ºC. ^{1}H NMR (300 MHz, DMSO-d_6) δ 9.47 (br, 2H), 7.83 (brs, 1H), 6.62 (d, *J* = 8.4 Hz, 2H), 6.49 (d, *J* = 8.4 Hz, 2H), 6.11 (d, *J* = 2.5 Hz, 1H), 6.04 (d, *J* = 2.2 Hz, 1H), 4.56 (brs, 2H), 3.79 (t, *J* = 6.5 Hz, 2H), 3.15 (t, *J* = 7.0 Hz, 2H), 2.13 (s, 3H), 1.61 (q, *J* = 7.0 Hz, 2H), 1.52 – 1.17 (m, 18H). ^{13}C NMR (126 MHz, $CDCl_3$) δ 170.6, 163.1, 158.8, 152.6, 139.2, 137.1,

116.8, 115.7, 110.6, 102.0, 68.7, 40.0, 29.48, 29.45, 29.38, 29.33, 29.29, 29.12, 26.99, 26.0, 22.9. MS (ESI^+) *m*/*z* 444 $[M+H]^+$. HRMS (ESI^+) *m/z* 442.2842 ($C_{26}H_{38}N_2O_4$ requires 442.2832).

Di-*tert*-butyl 2-((4-((12-(2,4-dihydroxy-6-methylbenzamido)dodecyl)oxy)phenyl)imino) imidazolidine-1,3-dicarboxylate (14c). Amine **12c** (50 mg, 0.113 mmol) and di-*tert*-butyl 2-thioxoimidazolidine-1,3-dicarboxylate **13** (37.6 mg, 0.124 mmol) were added in a Kimax tube and dissolved in 3 mL of anhydrous DMF under argon atmosphere. The reaction mixture was stirred at 0 ºC in an ice-water bath. Then, Et_3N (0.034 mL, 0.248 mmol) was added dropwise followed by the addition of $HgCl_2$ (33.7 mg, 0.124 mmol) in argon atmosphere. The reaction mixture was stirred 1 h at 0 ºC and next, it was brought to room temperature and stirred for 20 h. Finally, the suspension was filtered on celite and the solvent was evaporated under vacuum. The crude residue dissolved in CH_2Cl_2 and washed with an aqueous saturated solution of ammonium chloride. The organic phase was dried under vacuum to give a solid which was purified by radial chromatography with Hexane/EtOAc (50:50). Compound **14c** was obtained as reddish oil (69 mg, 88%). M.p. 46.1–49.5 ºC. ^1H NMR (500 MHz, $CDCl_3$) δ 6.92 (d, *J* = 8.9 Hz, 2H), 6.76 (d, *J* = 8.9 Hz, 2H), 6.28 (d, *J* = 2.6 Hz, 1H), 6.17 (d, *J* = 2.6 Hz, 1H), 5.94 (t, *J* = 5.9 Hz, 2H), 3.89 (t, *J* = 6.6 Hz, 2H), 3.81 (s, 4H), 3.45 (m, 2H), 2.44 (s, 3H), 1.73 (p, *J* = 6.9, 6.8 Hz, 2H), 1.60 (p, *J* = 7.3, 7.1 Hz, 2H), 1.55–1.31 (m, 34 H). ^{13}C NMR (126 MHz, $CDCl_3$) δ 170.6, 163.1, 159.2, 155.0, 150.5, 141.4, 138.9, 137.0, 122.5, 114.9, 110.8, 109.8, 82.8, 68.4, 43.2, 39.9, 29.7, 29.39, 29.36, 29.34, 29.32, 29.27, 29.21, 29.1, 28.0, 27.9, 27.0, 25.9, 22.9. HRMS (ESI^+) *m/z* 710.4247 ($C_{39}H_{58}N_4O_8$ requires 710.4255).

3) Synthesis of **19** and **20**

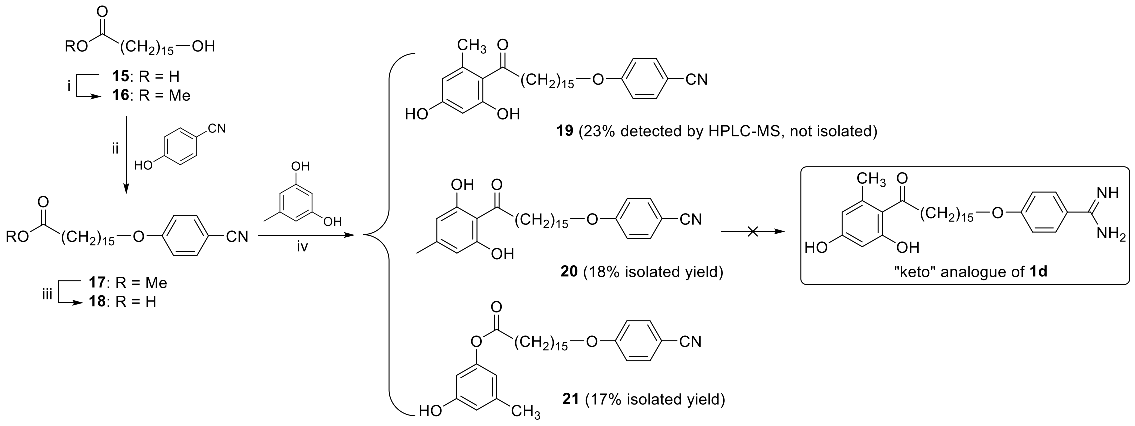

[a]Reagents and conditions: (i) MeOH, TsOH-H_2O, rt, 21 h, (96%); (ii) PPh_3, DIAD, THF, 0 ºC to rt, 4 days (58%); (iii) LiOH.H_2O, THF/MeOH/H_2O (2/1/1), rt (94%); (iv) $AlCl_3$, 1,2-dichloroethane, 100 ºC, 24 h.

Methyl 16-hydroxyhexadecanoate (16). A 250 mL round-bottomed flask was charged with 16-hydroxyhexadecanoic acid (1.52 g, 5.58 mmol) and *p*-toluenesulfonic acid monohydrate (317 mg, 1.84 mmol) in argon atmosphere at room temperature. Methanol (80 mL) was added and the reaction mixture was stirred for 21 h. The reaction was quenched with sodium bicarbonate (0.36 g, 4.29 mmol) and the reaction mixture was stirred 0.5 h. The suspension was filtered on celite and the solvent was evaporated under vacuum to yield **16** as white powdered solid (1.54 g, 96%). M.p. 54.8–28.8 ºC. ^{1}H NMR (300 MHz, $CDCl_3$) δ 3.65 (m, 4H), 2.30 (t, *J* = 7.5 Hz, 2H), 1.68 – 1.22 (m, 26H). ^{13}C NMR (126 MHz, $CDCl_3$) δ 174.4, 63.1, 51.5, 34.1, 32.8, 29.63, 29.62, 29.60, 29.58, 29.44, 29.26, 29.15, 25.7, 25.0.

Methyl 16-(4-cyanophenoxy)hexadecanoate (17). A solution of **16** (894 mg, 3.12 mmol), 4-cyanophenol (446 mg, 3.74 mmol) and triphenylphosphine (982 mg, 3.74 mmol) in anhydrous THF was stirred at 0 ºC in an ice-water bath. Then, diethyl azodicarboxylate (0.78 mL, 3.74 mmol) was added dropwise. After 0.5 h the reaction mixture was brought to room temperature and stirred for 4 days. The solvent was removed under vacuum and the crude product was partitioned between CH_2Cl_2 and water. Organic phase was washed with brine, dried ($MgSO_4$) and evaporated to give a yellow crude. Chromatography (50g SI) with Hexane/EtOAc (95:5 → 90:10) yielded **17** as colourless solid (674 mg, 58%). M.p. 82.7–83.8 ºC. ^{1}H NMR (500 MHz, $CDCl_3$) δ 7.57 (d, *J* = 9.0

Hz, 2H), 6.93 (d, J = 9.0 Hz, 2H), 3.99 (t, J = 6.5 Hz, 2H), 3.66 (s, 3H), 2.30 (t, J = 7.6 Hz, 2H), 1.79 (m, 2H), 1.61 (p, J = 7.40 Hz, 2H), 1.47 – 1.25 (m, 22H). ^{13}C NMR (126 MHz, $CDCl_3$) δ 174.4, 162.5, 134.0, 119.4, 115.2, 103.6, 68.4, 51.5, 34.1, 29.63, 29.59, 29.57, 29.53, 29.47, 29.45, 29.32, 29.26, 29.15, 28.98, 25.9, 25.0.

16-(4-cyanophenoxy)hexadecanoic acid (18). Compound **17** (620 mg, 1.6 mmol) and LiOH (153 mg, 6.4 mmol) were dissolved in a 2:1:1 mixture of THF:MeOH:H_2O (30 mL) in a round-bottomed flask. The reaction mixture was stirred at room temperature overnight, diluted with water (10 mL), and acidified with 1N HCl until pH = 2. The precipitate was collected by filtration to yield **18** as white solid (561.5 mg, 94%). M.p. 104.3–109.4 ºC. ^{1}H NMR (500 MHz, DMSO-d_6) δ 11.95 (br s, 1H), 7.75 (d, J = 9.0 Hz, 2H), 7.09 (d, J = 9.0 Hz, 2H), 4.04 (t, J = 6.5 Hz, 2H), 2.18 (t, J = 7.4 Hz, 2H), 1.70 (m, 2H), 1.47 (p, J = 7.0 Hz, 2H), 1.41 – 1.22 (m, 22H). ^{13}C NMR (126 MHz, $CDCl_3$) δ 174.4, 162.5, 134.0, 119.4, 115.2, 103.6, 68.4, 51.5, 34.1, 29.63, 29.59, 29.57, 29.53, 29.47, 29.45, 29.32, 29.26, 29.15, 28.98, 25.9, 25.0.

4-((16-(2,6-dihydroxy-4-methylphenyl)-16-oxohexadecyl)oxy)benzonitrile (20). An oven-dried round-bottomed flask with a screw cap was charged with **18** (750 mg, 1.94 mmol) in anhydrous 1,2-dichloroethane (15 mL). 5-Methylresorcinol (356 mg, 2.87 mmol) and aluminium chloride (382 mg, 2.87 mmol) in anhydrous 1,2-dichloroethane (15 mL) were added dropwise to the reaction mixture at 50 ºC. The reaction mixture was refluxed at 100 ºC for 24 h. The flask was cooled to room temperature, and 20 mL of water were poured into the reaction mixture followed by acidification with HCl 0.1 M. Extraction with EtOAc was performed three times. The organic phase was washed with water (2×) brine (2×), dried ($MgSO_4$), and evaporated to give a brown crude. Chromatography (15g SI) with Toluene/EtOAc (100:0→ 95:5) yielded **20** as a yellow solid (177 mg, 18%). HPLC–MS (UV) > 98%. M.p. 89.7–94.7 ºC. ^{1}H NMR (500 MHz, $CDCl_3$) δ 9.66 (s, 1H), 7.57 (d, J = 8.8 Hz, 2H), 6.93 (d, J = 8.8 Hz, 2H), 6.21 (s, 2H), 3.99 (t, J = 6.5 Hz, 2H), 3.09 (t, J = 7.4 Hz, 2H), 2.23 (s, 3H), 1.78 (m, 2H), 1.69 (p, J = 7.5 Hz, 2H), 1.48 – 1.25 (m, 22H). ^{13}C NMR (126 MHz, $CDCl_3$) δ 206.3, 161.5, 160.2, 146.3, 133.0, 118.3, 114.18, 114.20, 108.1, 106.9, 102.4, 67.4, 43.5, 28.63, 28.61, 28.58, 28.54, 28.51, 28.47, 28.42, 28.25, 24.9, 23.5, 20.8. HRMS (ESI^+) m/z 479.3036 ($C_{30}H_{41}NO_4$ requires 479.3036).

3-hydroxy-5-methylphenyl 16-(4-cyanophenoxy)hexadecanoate (21) was obtained as a secondary product. Yellow solid (161.6 mg, 17%). HPLC–MS (UV) > 98%. M.p. 94.0–

99.3 ºC. ^{1}H NMR (500 MHz, $CDCl_3$) δ 7.57 (d, *J* = 8.9 Hz, 2H), 6.98 (d, *J* = 8.9 Hz, 2H), 6.50 (s, 1H), 6.46 (s, 1H), 6.39 (s, 1H), 3.99 (t, *J* = 6.5 Hz, 2H), 2.52 (t, *J* = 7.5 Hz, 2H), 2.28 (s, 3H), 1.76 (m, 4H), 1.39 – 1.26 (m, 22H). ^{13}C NMR (126 MHz, $CDCl_3$) δ 171.5, 161.5, 155.3, 150.4, 139.6, 133.0, 124.5, 118.3, 114.2, 113.5, 112.7, 105.2, 102.5, 67.4, 33.4, 29.3, 28.68, 28.61, 28.60, 28.56, 28.52, 28.49, 28.42, 28.27, 28.23, 28.07, 27.94, 24.9, 23.9, 20.3. HRMS (ESI^+) *m/z* 479.3036 ($C_{30}H_{41}NO_4$ requires 479.3036).

Compound **1a**

^{1}H NMR (400 MHz, DMSO-d_6)

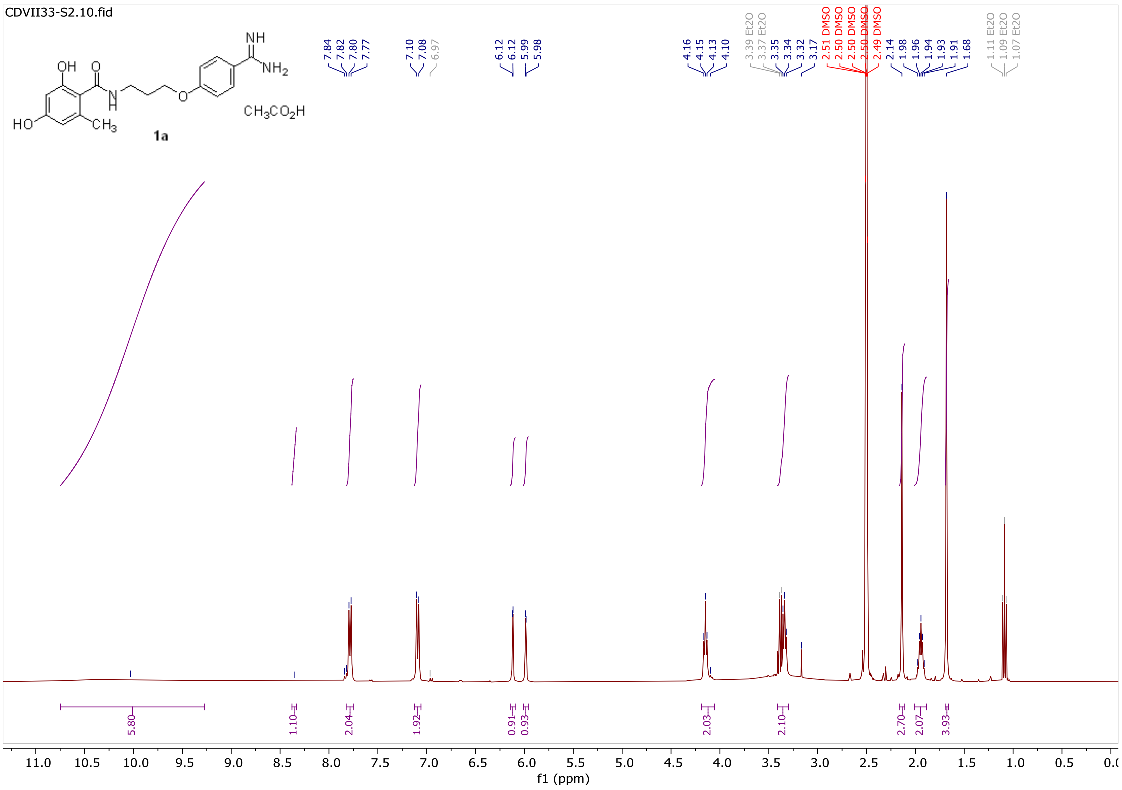

^{13}C NMR (101 MHz, DMSO-d_6)

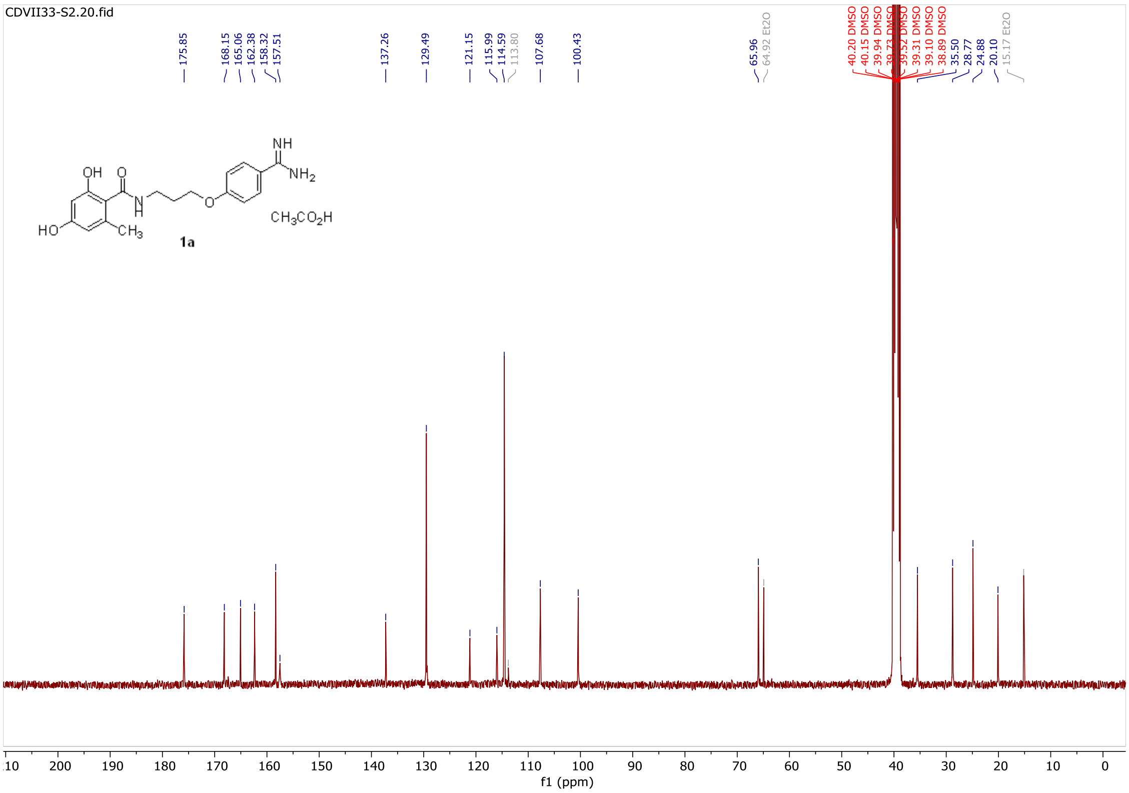

Compound **1b**

^{1}H NMR (500 MHz, DMSO-d_6)

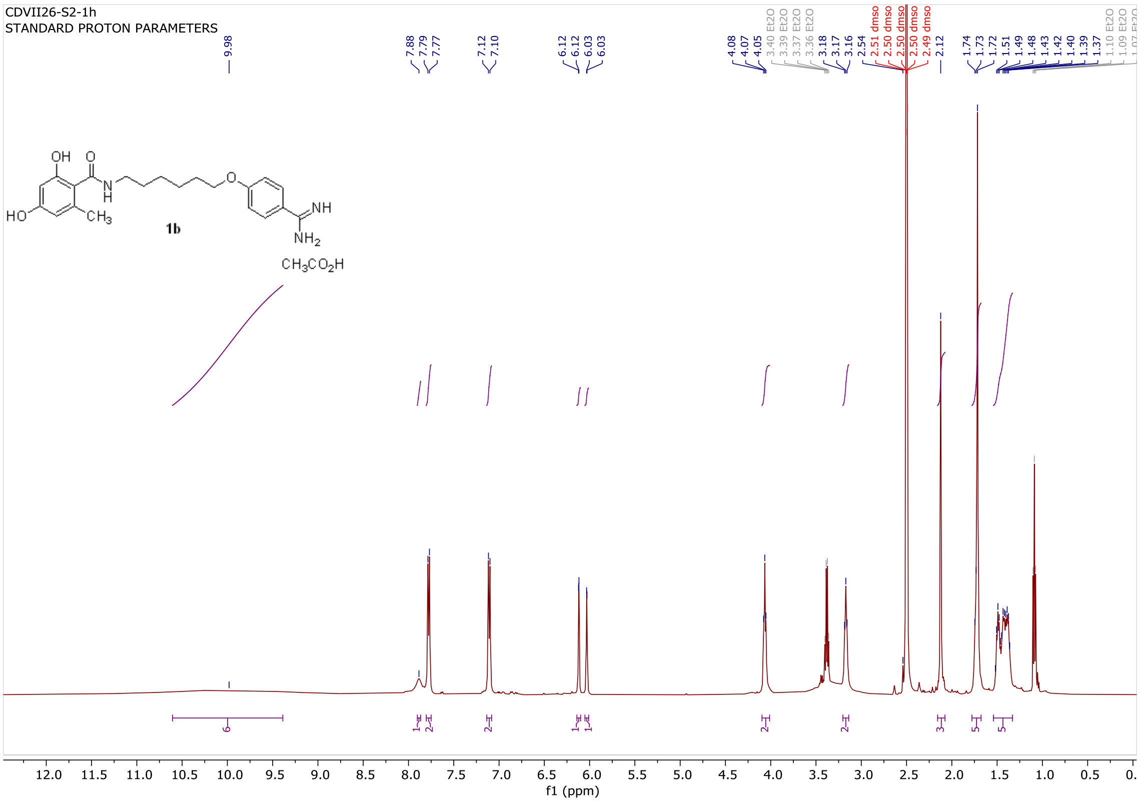

^{13}C NMR (126 MHz, DMSO-d_6)

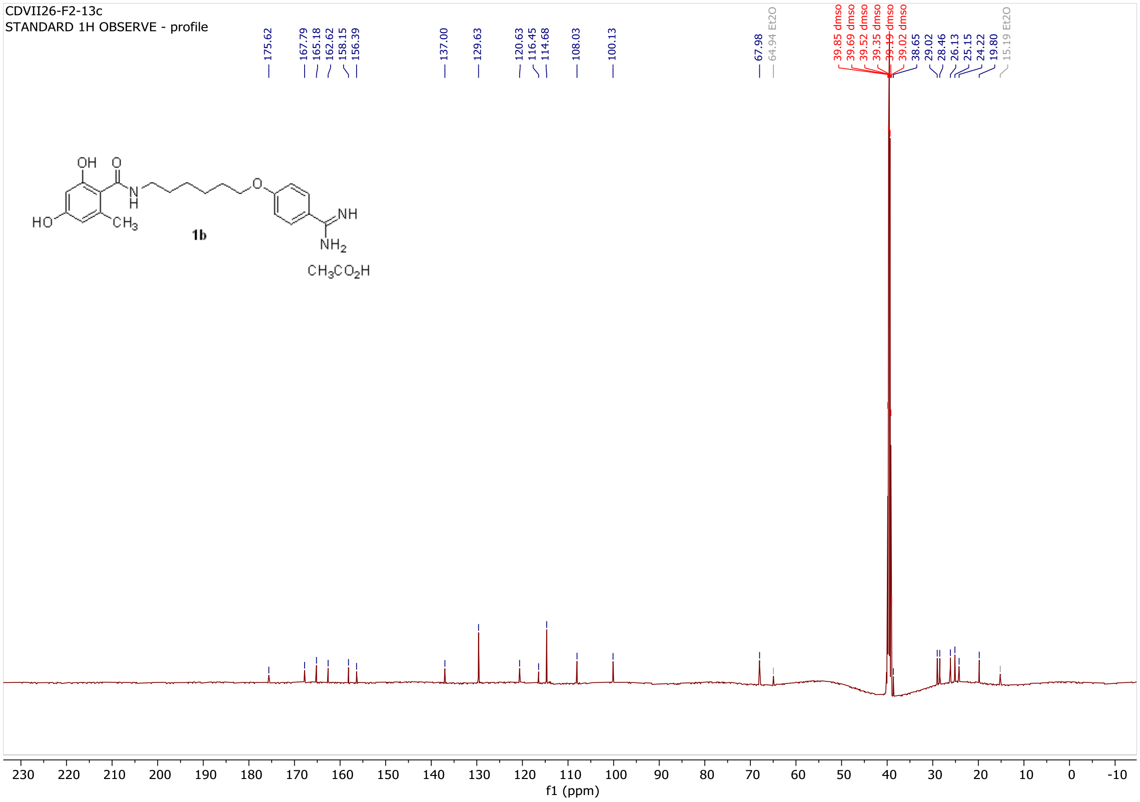

Compound **1c**

^{1}H NMR (300 MHz, DMSO-d_6)

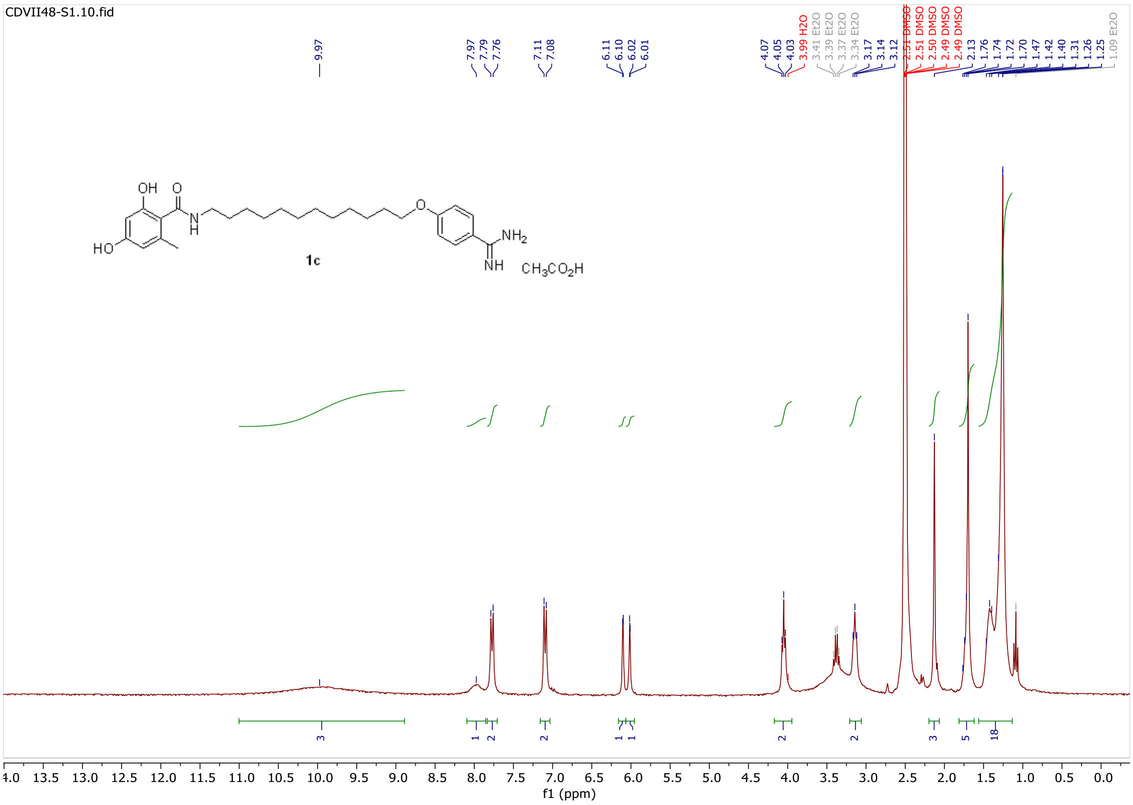

^{13}C NMR (101 MHz, DMSO-d_6)

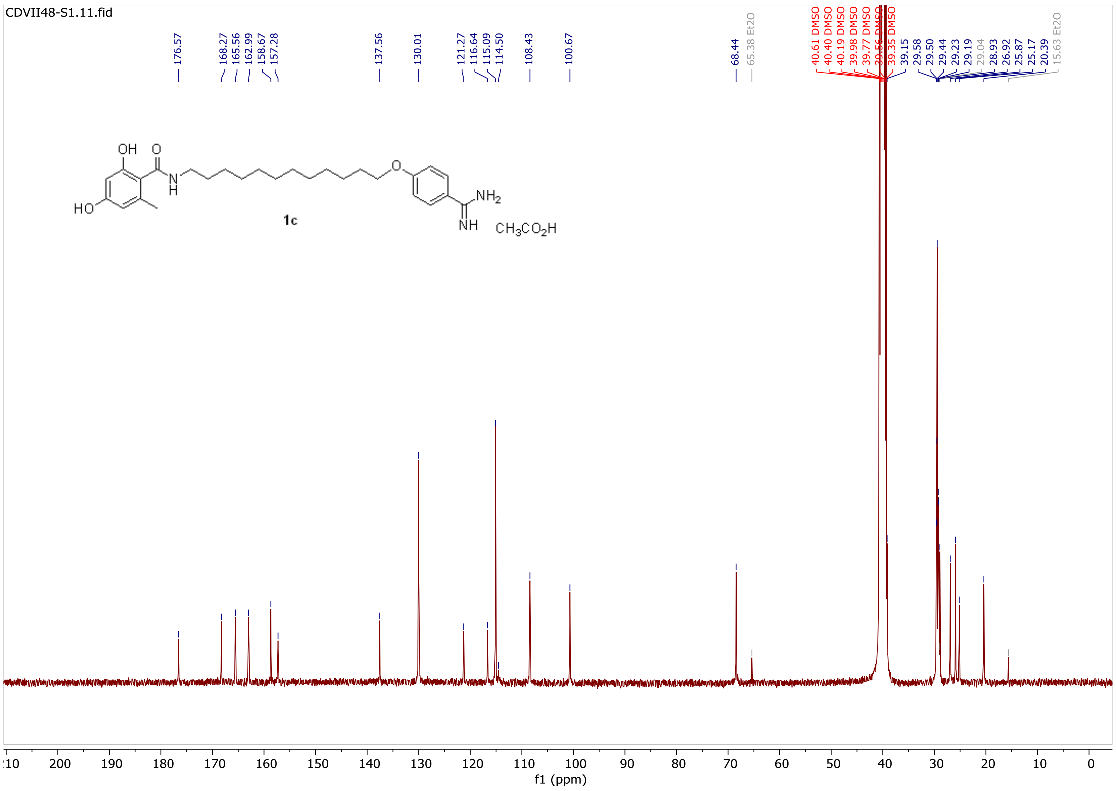

Compound **1d**

^{1}H NMR (400 MHz, DMSO-d_6)

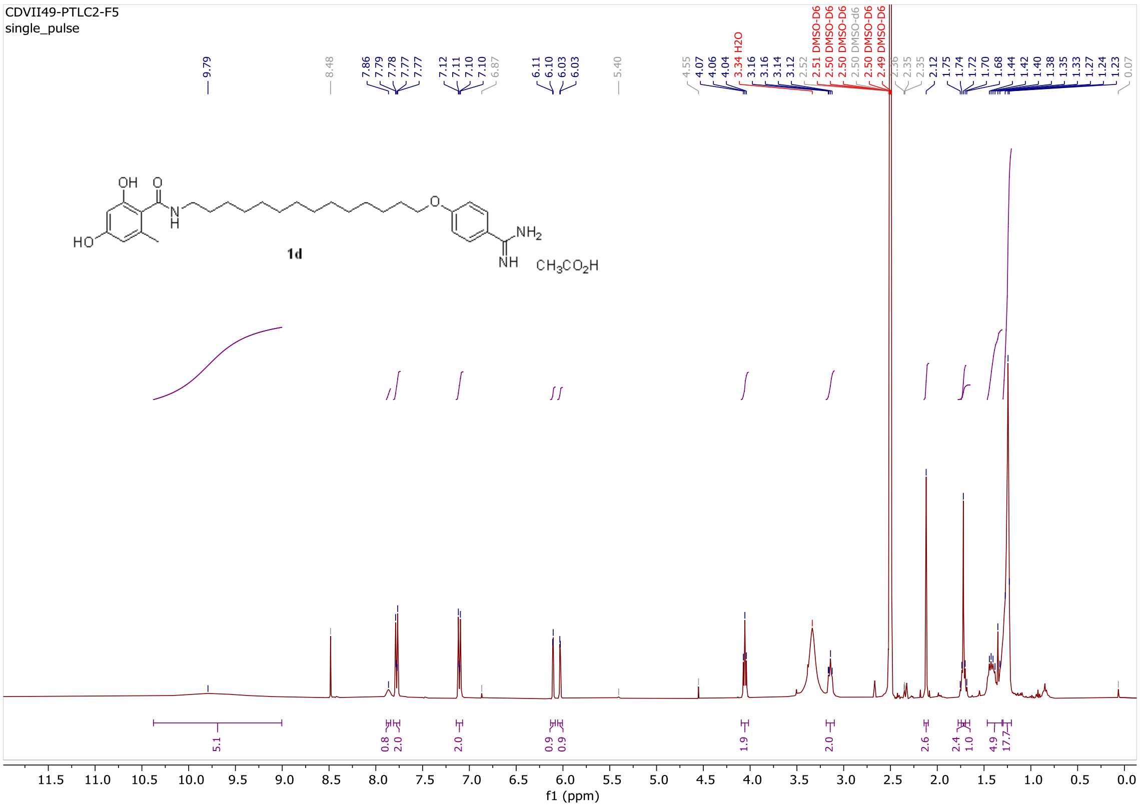

^{13}C NMR (101 MHz, DMSO-d_6)

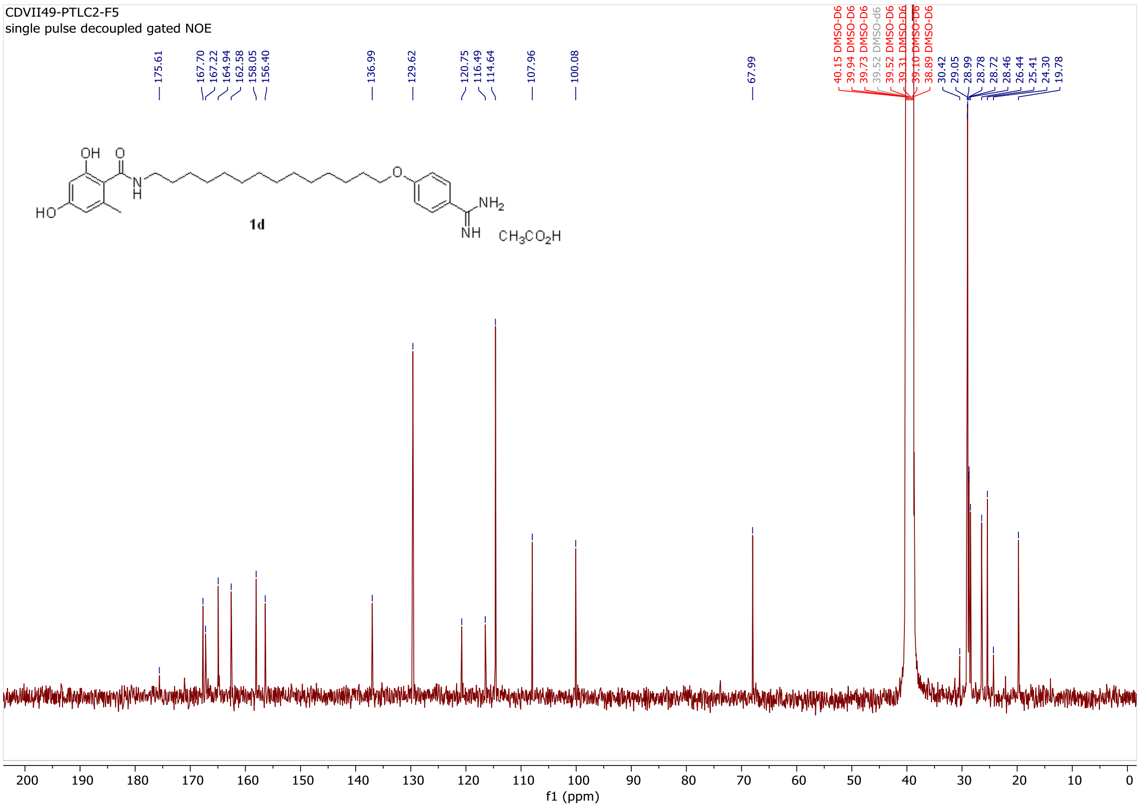

Compound **2c**

^{1}H NMR (500 MHz, Methanol-d_4)

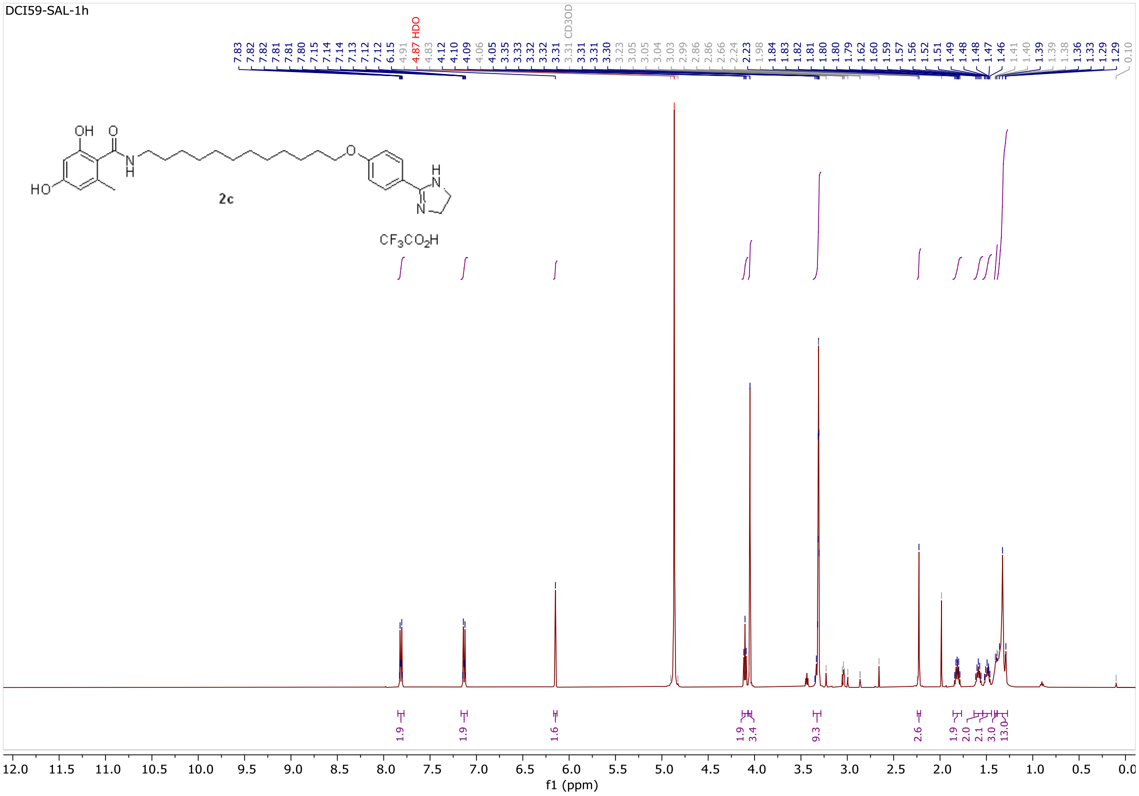

^{13}C NMR (101 MHz, Methanol-d_4)

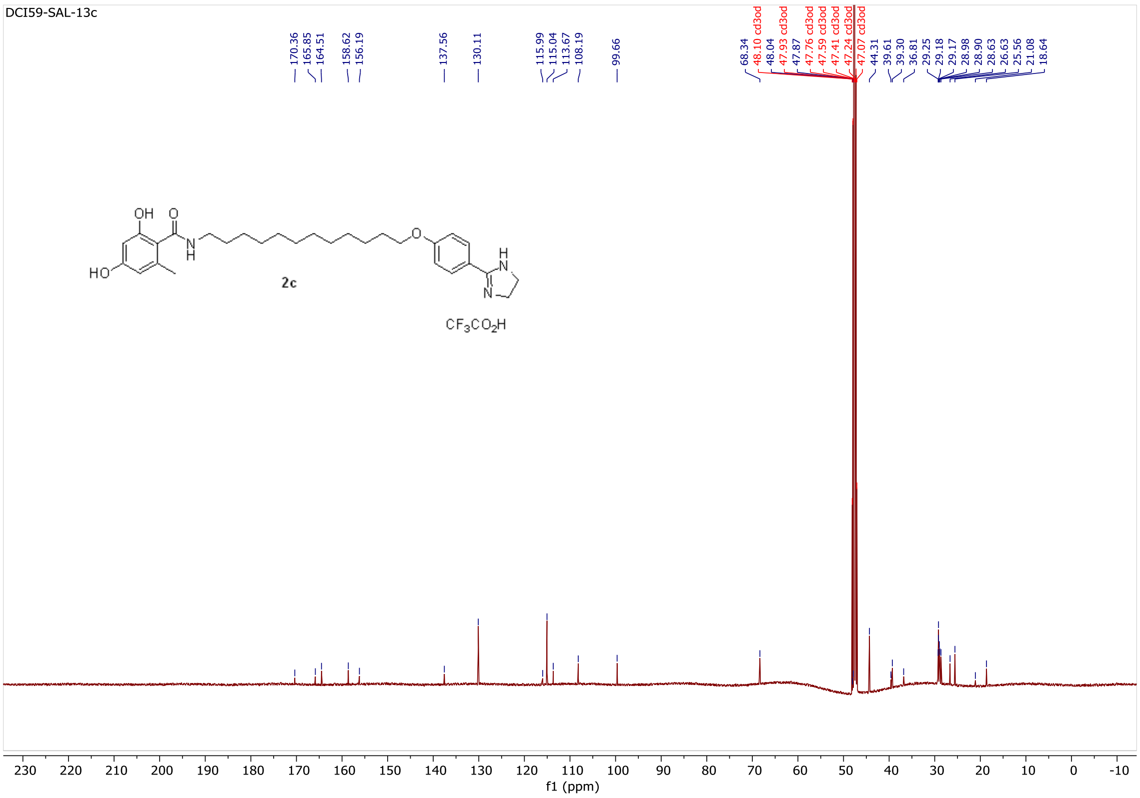

Compound **3c**

^{1}H NMR (500 MHz, Methanol-d_4)

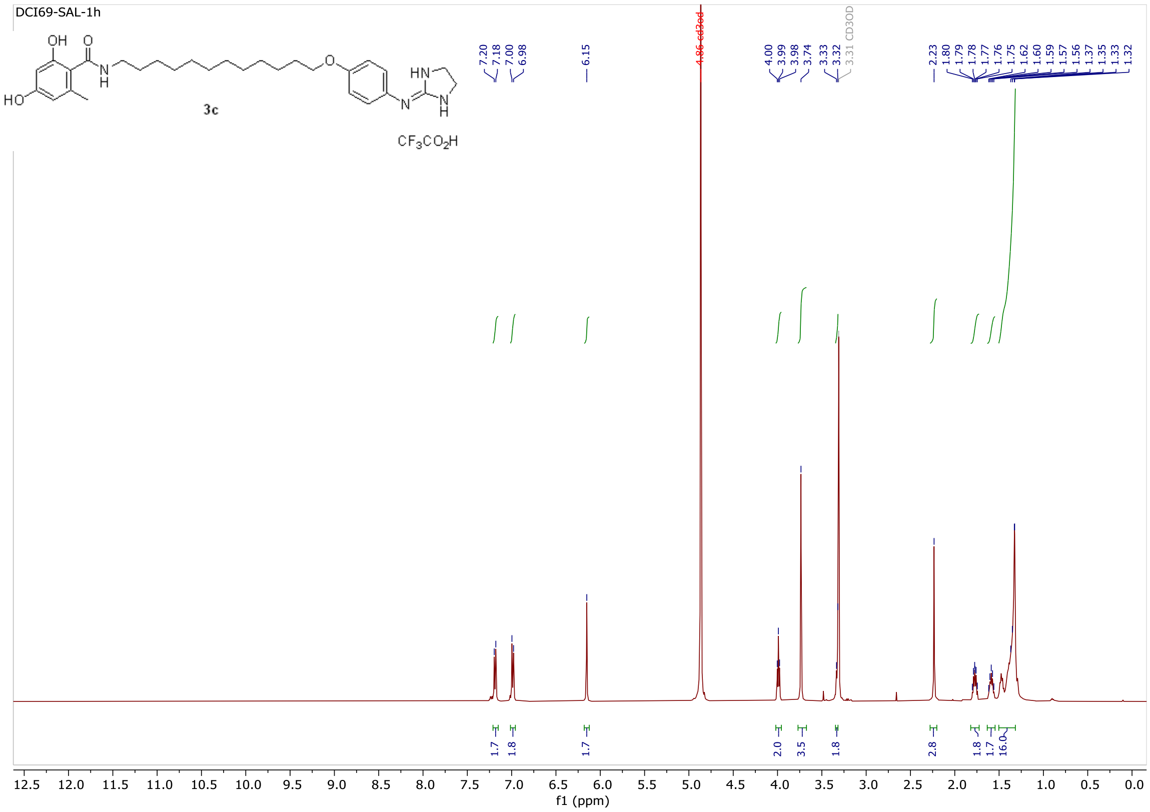

^{13}C NMR (101 MHz, Methanol-d_4)

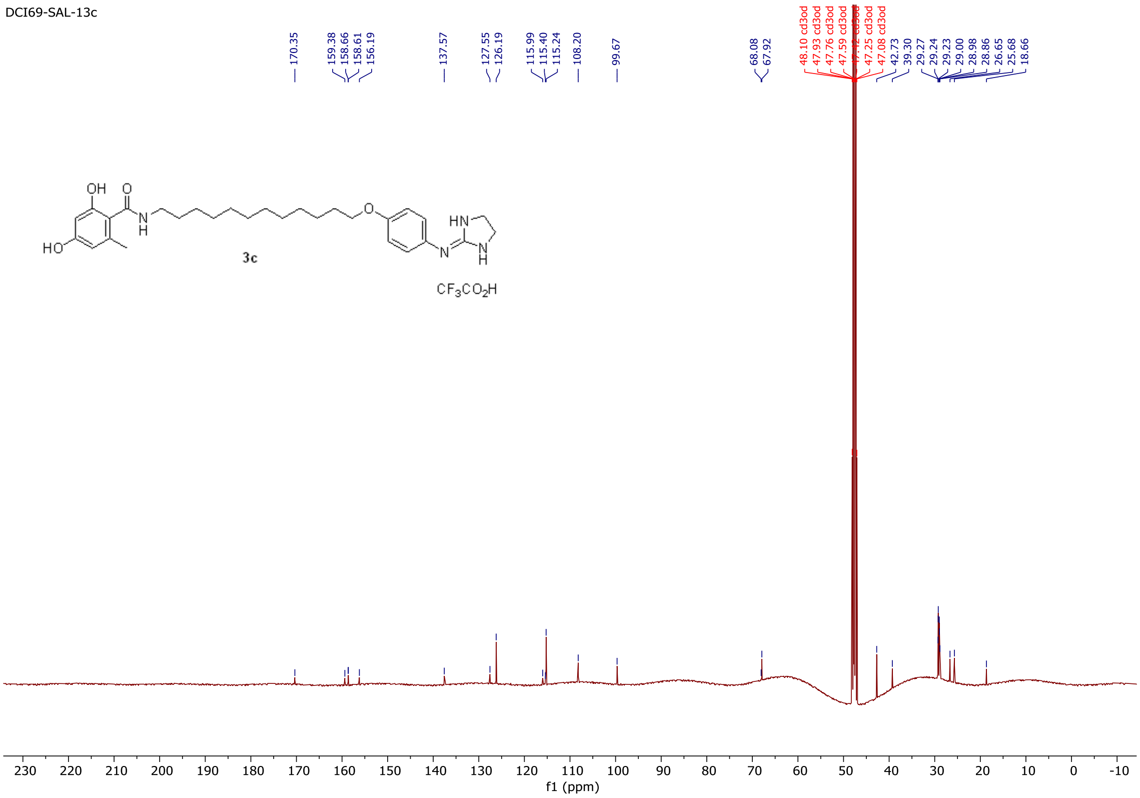

Compound **8a**

^{1}H NMR (300 MHz, DMSO-d_6)

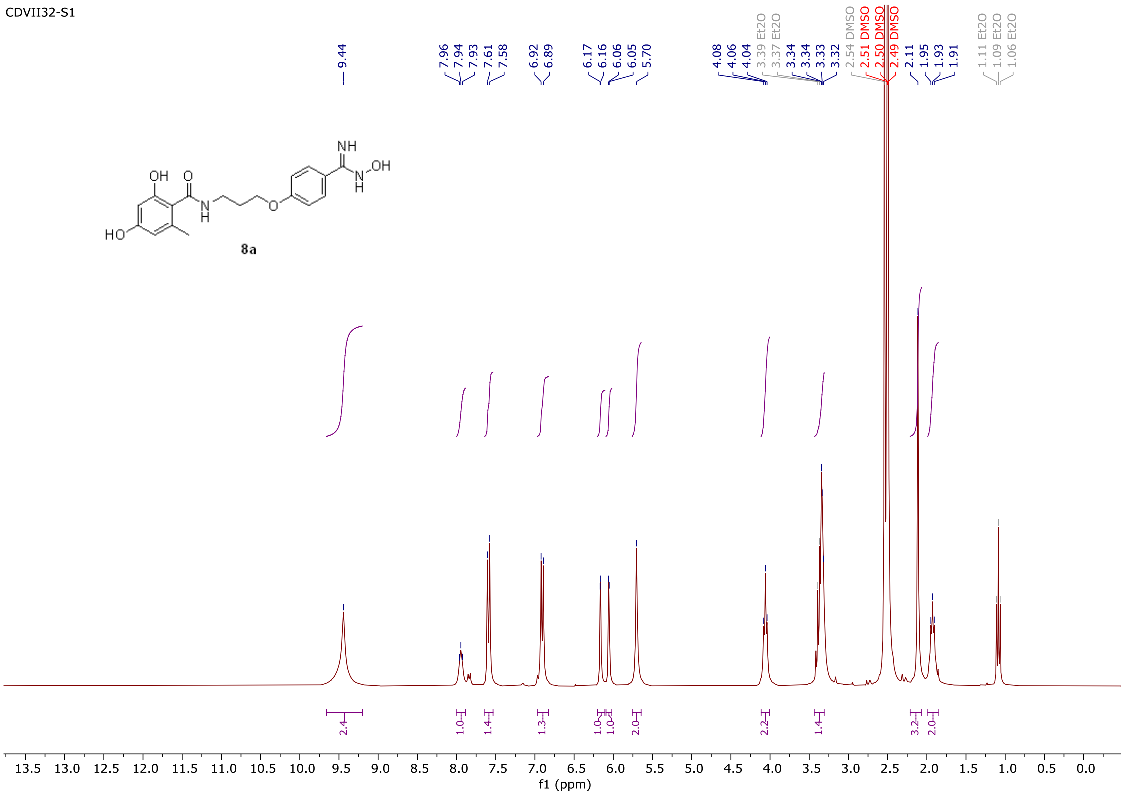

^{13}C NMR (75 MHz, DMSO-d_6)

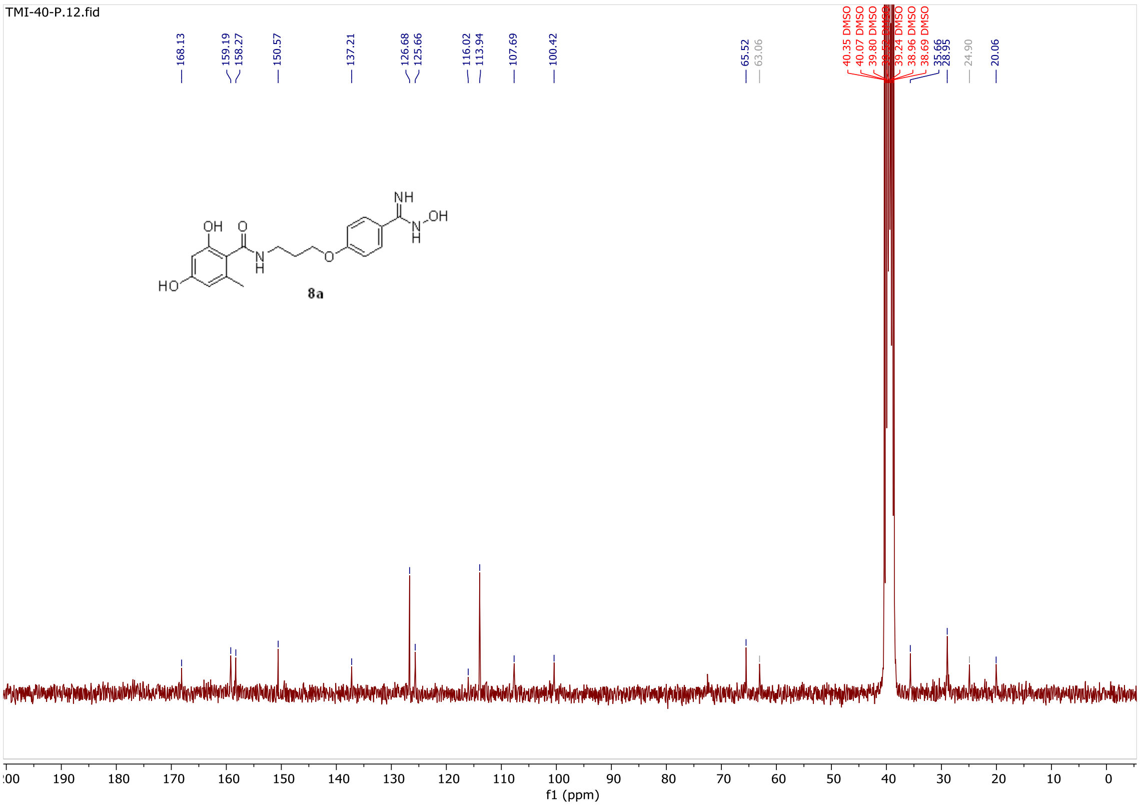

Compound **8b**

^{1}H NMR (500 MHz, Methanol-d_4)

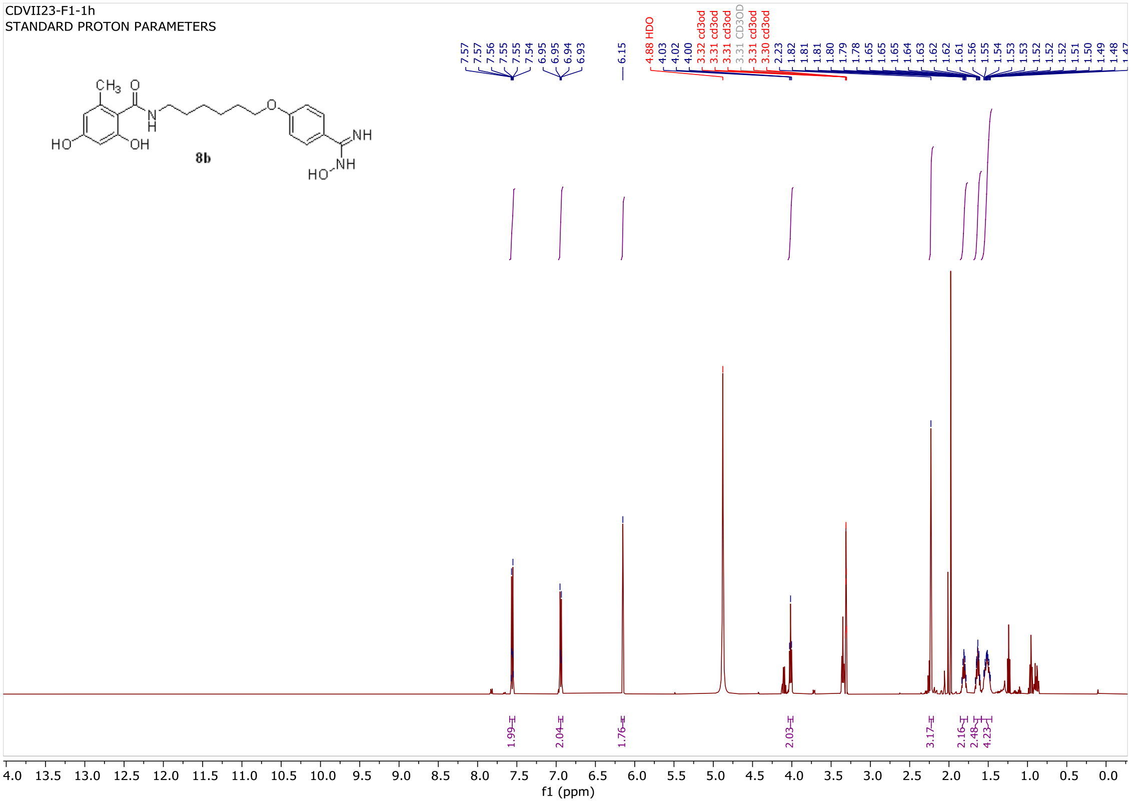

Compound **8c**

^{1}H NMR (300 MHz, DMSO-d_6)

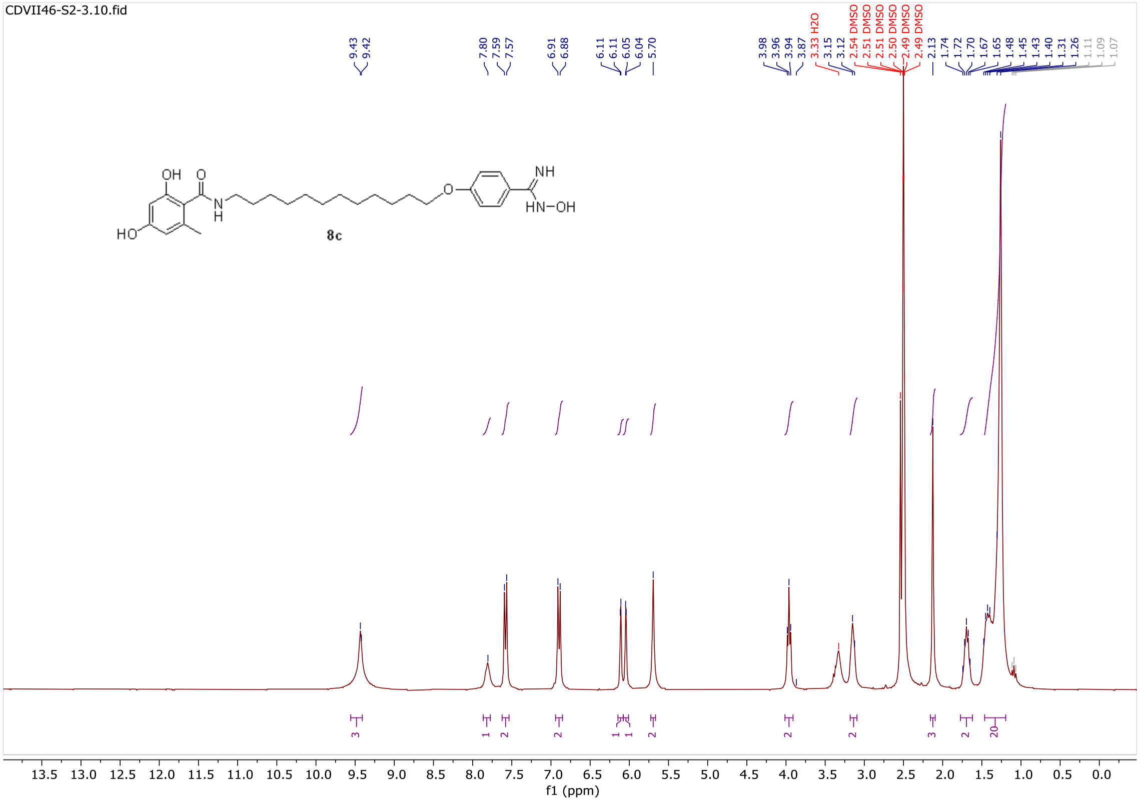

^{13}C NMR (75 MHz, DMSO-d_6)

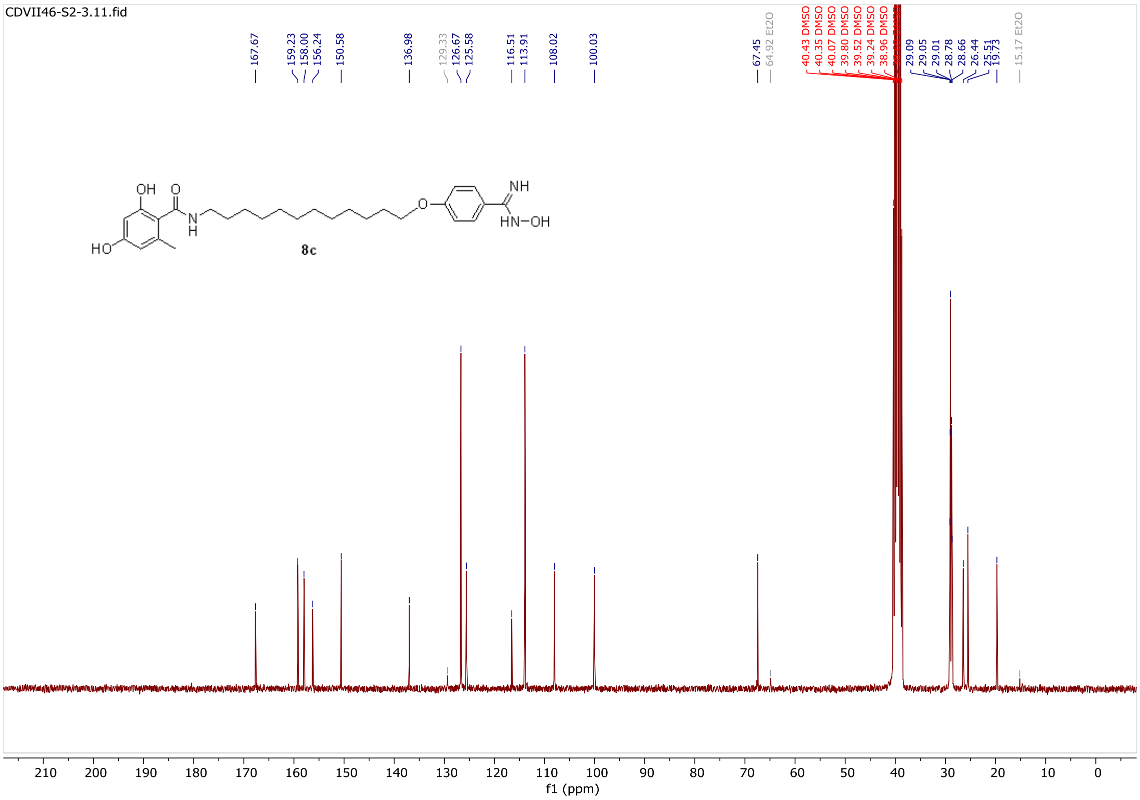

Compound **8d**

^{1}H NMR (400 MHz, Methanol-d_4)

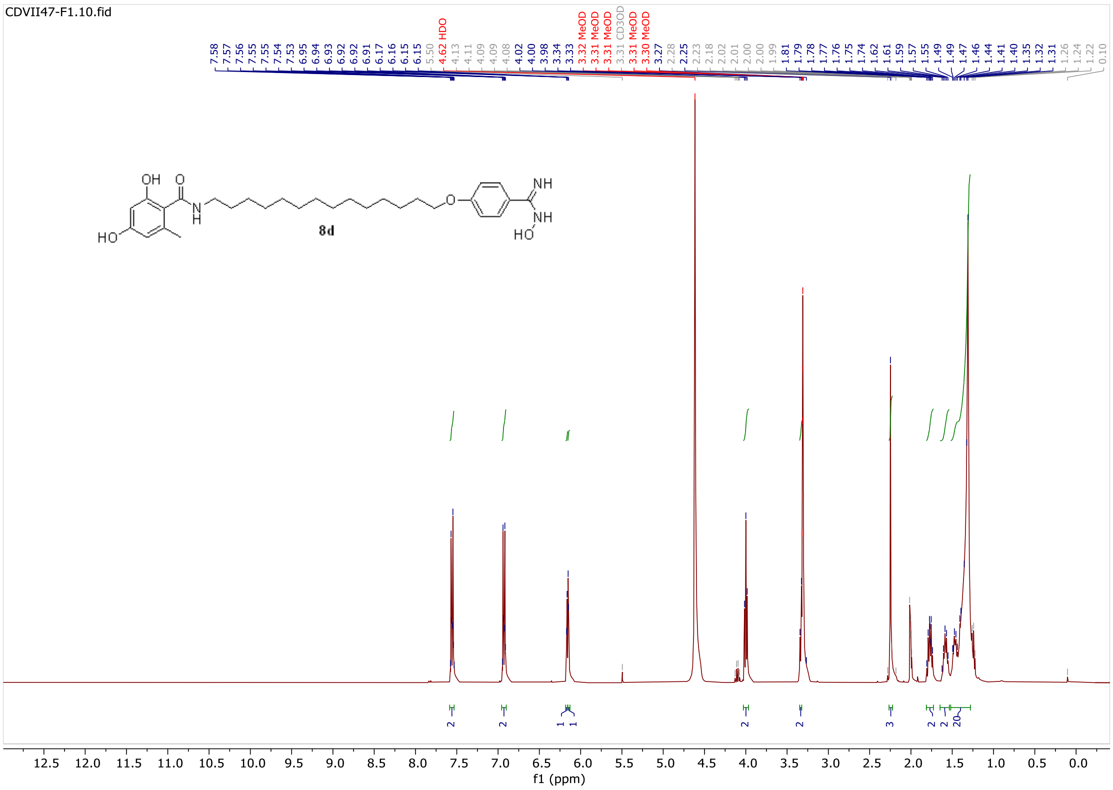

^{13}C NMR (100 MHz, Methanol-d_4+CD_3CN)

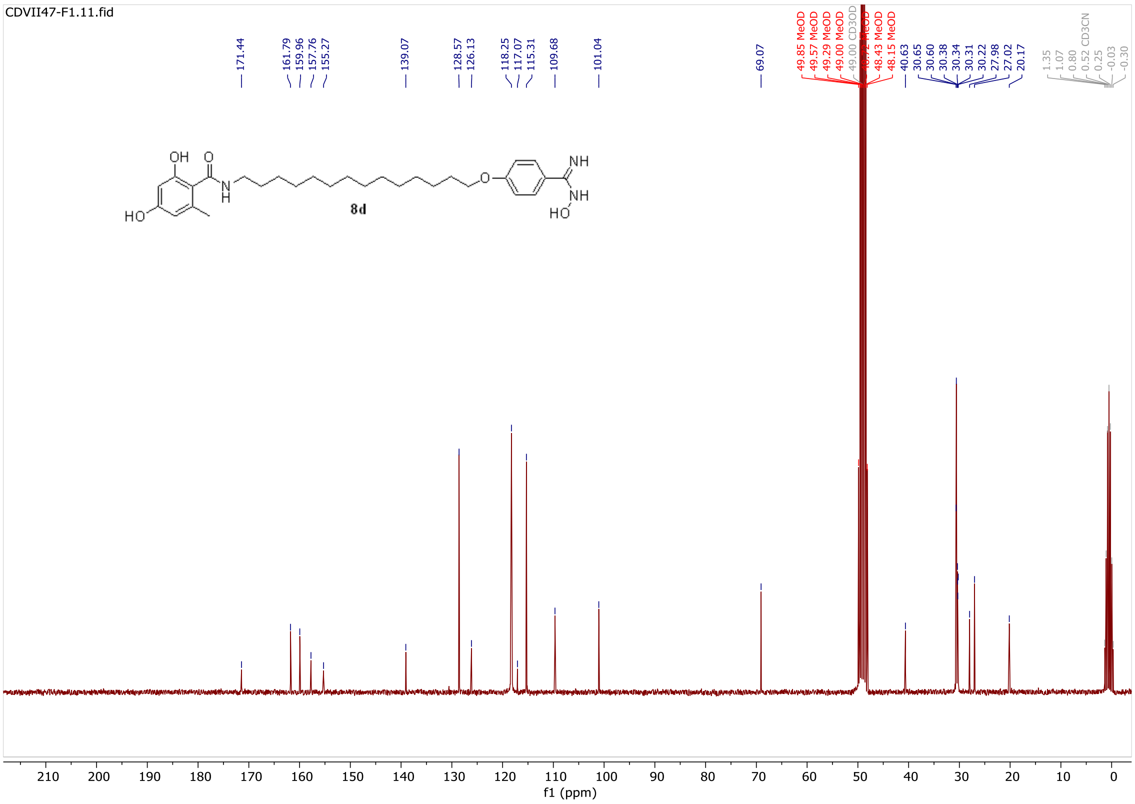

Supplement: Supplementary file 1 — ml1c00717_si_001.pdf [file ml1c00717_si_001.pdf]
